# Early-life thymectomy results in visceral adipose tissue inflammation and glucose intolerance

**DOI:** 10.1186/s12979-025-00531-x

**Published:** 2025-10-01

**Authors:** David J. Buckley, Sogol Zahedi, Prema Velusamy, Sunita Sharma, Manoj Sabnani, Lisa C. Flores, Ganga Tandukar, Yuji Ikeno, Paul J. Fadel, Lisa A. Lesniewski, Daniel W. Trott

**Affiliations:** 1 Department of Kinesiology, University of Texas Arlington, Arlington, TX USA; 2 Barshop Institute for Longevity and Aging Studies, The University of Texas Health Science Center at San Antonio, San Antonio, TX USA; 3 Department of Pathology and Laboratory Medicine, The University of Texas Health Science Center at San Antonio, San Antonio, TX USA; 4 Geriatric Research and Education Center, Audie L. Murphy VA Hospital South Texas Veterans Health Care System, San Antonio, TX USA; 5 Department of Nutrition and Integrative Physiology, The University of Utah, Salt Lake City, Utah USA; 6 Division of Geriatrics, Department of Internal Medicine, The University of Utah School of Medicine, Salt Lake City, Utah USA; 7 Nora Eccles Harrison Cardiovascular Research and Training Institute, The University of Utah, Salt Lake City, Utah USA; 8 Geriatric Research Education and Clinical Center, Veteran’s Affairs Medical Center-Salt Lake City, Salt Lake City, Utah USA

**Keywords:** Aging, Thymectomy, T cell, Glucose intolerance, Inflammation

## Abstract

We have previously demonstrated that proinflammatory T cells in adipose tissue and the liver play a mechanistic role in glucose intolerance in old mice. Further, we and others have demonstrated that early life thymectomy results in a T cell phenotype that shares many features of classical T cell aging in an otherwise young healthy mouse. In this investigation, we sought to test the hypothesis that inducing premature T cell aging via early life thymectomy results in T cell mediated inflammation of the liver and visceral adipose tissue, as well as glucose intolerance in otherwise young mice. Mice were thymectomized at three weeks of age. At 9 months of age, thymectomized mice exhibited glucose intolerance that was independent of body mass along with greater frailty. Thymectomized mice exhibited blunted proportions of naïve and greater proportions of memory cells in the spleen, liver and perigonadal adipose tissue (pgWAT). Bulk RNAseq of the pgWAT revealed that thymectomized mice exhibited an upregulation of genes responsible for immune activation, chemokine signaling, and inflammation along with a downregulation of genes responsible for metabolic function. We also found that T cells in the pgWAT of thymectomized mice exhibited greater chemokine receptor expression as well as increased markers of histopathological inflammation that were independent of greater adipose tissue expansion. These results suggest that early life thymectomy results in T cell mediated pgWAT inflammation, systemic glucose intolerance and frailty in adult mice.

**Supplementary Information:**

The online version contains supplementary material available at 10.1186/s12979-025-00531-x.

## Background

Impaired glucose homeostasis is characterized by glucose intolerance and insulin resistance [1]. Furthermore, impaired glucose homeostasis is associated with frailty and disease burden in older adults [2–5]. The mechanisms that underlie the disruption of glucose homeostasis are multi-faceted and are associated with the dysfunction of several cell types and tissues, including the perigonadal white adipose tissue (pgWAT) and liver [6]. T cells have been implicated in dysfunction of the pgWAT and liver resulting in subsequent glucose intolerance in diet induced obesity, aging, and models of pre-mature senescence [7–16]. However, it is unknown whether aged T cells *alone* are sufficient to induce liver and pgWAT inflammation and subsequent glucose intolerance in an otherwise young animal.

Previously, we have demonstrated that old (22-24mo) mice have greater numbers of T cells in the pgWAT and liver compared to young (4-6mo) mice [7]. When we pharmacologically depleted pan (CD3 +) T cells, we observed that old T cell depleted mice exhibited improved glucose tolerance compared to T cell intact mice [7]. Similarly, others have demonstrated that treatment with the senolytic drugs dasatinib and quercetin results in blunted T cell infiltration and inflammation in the pgWAT and liver as well as improvements in systemic metabolism in old mice [8]. These studies support a major role of aged T cells in age-related metabolic impairments; however, whether aged T cells alone can induce inflammation of the liver and pgWAT and subsequent impairments in glucose homeostasis is less clear.

One major feature of T cell aging is thymic involution [17]. The thymus is responsible for the development of new naïve T cells as well as the removal of T cells that may be self-reactive [18–20]. In humans, the epithelium of the thymus is slowly replaced by adipose tissue beginning at sexual maturity resulting in decreased naïve T cell output and a subsequent decrease in the proportion of naïve T cells. Thymic involution is well known to contribute to impaired immune function with age but whether thymic involution and impaired T cell function may also play a role in the development of metabolic impairments is less clear. In humans, neonatal thymectomy induces a decline in naïve T cells, resulting in a contracted T cell repertoire and greater oligoclonal expansion, all of which are consistent with T cell aging [21–23]. In the current study, we sought to leverage the observation that in the absence of a functional thymus (thymectomy), T cells undergo homeostatic proliferation to maintain the T cell pool. This results in a T cell population skewed toward a memory phenotype, similar to what occurs with aging. This thymectomized mouse model results in a young animal with aged-like T cell populations [24–29]. We have previously found that adult mice that were thymectomized at 3 weeks of age exhibit a T cell phenotype (e.g. shift from naïve towards memory) that is similar to old (22-24mo) mice [24]. These mice also exhibit greater large artery stiffness and endothelial dysfunction [24]. This investigation provides proof of concept that aged T cells alone can drive decline of other organs even in the absence of age, diet induced obesity, or genetic alterations to their immune system [24]. However, it is unknown whether the induction of aged T cells via early life thymectomy is sufficient to induce inflammation of the liver and pgWAT and ultimately glucose intolerance. Therefore, we sought to test the hypothesis that early life thymectomy results in T cell mediated inflammation of the liver and pgWAT, as well as glucose intolerance in otherwise young mice.

## Methods and materials

### Animals

All animal experiments conformed to the Guide and Use of Laboratory Animals and were approved by the University of Texas at Arlington Animal Care and Use Committees. All experiments utilized male C57BL/6 mice obtained from Charles River Inc. Mice were housed in environmental conditions (68–78°F; 30–70% humidity) consistent with the *Guide for the care and use of laboratory animals 8th edition* [30]*.* At three weeks of age, mice were thymectomized or left with their thymus intact (control) and euthanized at 9 months of age. All mice were housed under specific pathogen-free conditions in standard mouse cages on a 12:12 light:dark cycle with ad libitum access to food (Teklad 2018 diet) and water in the animal facility at the University of Texas at Arlington. Mice were fasted 2 h prior to euthanasia by terminal cardiac puncture followed by secondary bilateral thoracotomy while under isoflurane anesthesia. Absence of the thymus in thymectomized mice was confirmed at the time of euthanasia.

### Glucose and insulin tolerance testing

Glucose tolerance (GTT) and Insulin tolerance (ITT) was assessed one week apart at 6 months and 9 months of age as previously described [7, 8, 31]. Briefly, the mice were fasted for 6 h in the morning. Following the fast period, baseline glucose was measured in blood collected via a tail nick using a Contour nextOne handheld blood glucose monitor. Mice were then injected intraperitoneally with glucose (2 g/kg body mass, ip) for the GTT or Insulin (0.75 IU/kg body mass, ip), and blood glucose level was measured again 15, 30, 60, 90, and 120 min after the injection. Additionally, for the GTT a 15-μl blood sample was collected before the injection to measure baseline insulin.

### Frailty

In order to assess frailty, we used a novel 31-item frailty index based on established clinical signs of deterioration in mice 2 h prior to euthanasia, as previously described [32–34]. Briefly, the frailty assessment evaluates the integumentary, musculoskeletal, vestibulocochlear/auditory, ocular and nasal, digestive, urogenital, and respiratory systems, while also looking for signs of discomfort. In addition, body weight and temperature were recorded. Scores were rated on a 3 point scale where a score of “0” represents absent, “0.5” represents mild, and a score of “1” represents severe using a score card and reference sheet [32]. The scores for a single animal were then calculated and reported.

### Enzyme-linked immunosorbent assay

Fasted (6 h) plasma insulin was measured using a commercially available rat/mouse insulin ELISA kit (Crystal Chem) according to the manufacturer’s protocol.

### Flow cytometry

Flow cytometry was performed as previously described [7, 24, 35]. Immediately following euthanasia, to remove circulating leukocytes from tissues, the chest cavity was opened, and the right atrium was nicked. A cannula was placed in the left ventricle and the animals were perfused with saline at physiological pressure until the effluent was cleared of blood and the liver was pale. Spleens, pgWAT, and a lobe of liver were excised, weighed, and digested using collagenase type I (Worthington, 450 U ml^−1^), and DNAse (Worthington, 0.1 mg ml^−1^) dissolved in DPBS buffer containing calcium and magnesium for 30 min at 37 °C. The tissues were further dispersed using repeated pipetting and the resultant homogenate was passed through a 70 μm sterile filter, yielding single-cell suspensions. Red blood cells were lysed by incubating cell suspensions in Ammonuim-Chloride-Potassium buffer for 5 min. Flow cytometry was performed on either a Cytek Northern Lights Flow Cytometer or BD FACSMelody. Dead cells were labelled with Tonbo Ghost Dye and excluded from analysis. Single-cell suspensions from Spleen, pgWAT, and liver were labelled with the following anti-mouse antibodies: Northern Lights Panel: AlexaFluor532 CD45, Invitrogen#59–0454-82 (total leukocytes), violetfluor500 CD3, Tonbo#85–0032 (pan T cells), BV570-CD4, Biolegend#100542 (T helper cells), PerCP-Cy5.5-CD8, Tonbo#65–0081 (cytotoxic T cells), SB645 CD44, Invitrogen#64–0441-82 (naïve vs. memory) and SB600 -CD62L Invitrogen#63–0621-82 (central vs.effector), PE CD49d Biolegend#103705 (virtual memory; defined as CD44^Hi^ CD49d^Lo^ [36, 37]), PE/Cyanine7 Biolegend#150612 CCR2, Alexa Fluor 488 Biolegend#107008, Super Bright 436 Invitrogen#62–1831-82 CXCR3. BD FACSMelody Panel: PE594 CD45, Biolegend#109846 (total leukocytes), APC CD3, Tonbo#20–0031 (pan T Cells), FITC CD4 Tonbo#35–0042 (T Helper Cells), PerCP-Cy5.5 CD8, Tonbo#65–0081 (cytotoxic T cells), PE-Cy5 CD44, Tonbo#55–0081, and (naïve vs memory), PE-Cy7 CD62L. Tonbo#60–0621, (central vs effector). Optimal antibody concentrations were determined by preliminary titration experiments. Gating strategy is presented in Supplemental Fig. 1–3. All flow cytometry data was collected at euthanasia when mice were 9-months old.

### T cell proliferation & multiplex cytokine bead kit

Using negative selection kits (Stem Cell Technologies), 50,000 CD4 + or CD8 + T cells were isolated and stimulated with anti-CD3 and anti-CD28 beads and allowed to proliferate for 96 h. At 96 h, the CD4 + and CD8 + T cells were counted, and the media was collected. We then assessed media cytokine concentrations using a Mouse Multiplex Cytokine Bead Kit (Biolegend) according to the manufacturer’s protocols. To account for differences in the total number of cells after 96 h, cytokine concentrations were normalized to pg/mL per 10^5^ cells.

### RNAseq

A pgWAT fat pad and a lobe of liver from thymectomized and control mice were homogenized and RNA was extracted using the Trizol method. Sample quality control was performed using an Agilent bioanalyzer and a library was created using a NEBNext Ultra II RNA Library Prep by Illumina and a NovaSeq 6000 S4 (PE150) sequencer. Messenger RNA was purified from total RNA using poly-T oligo-attached magnetic beads. After fragmentation, the first strand cDNA was synthesized using random hexamer primers, followed by the second strand cDNA synthesis using dTTP for non-directional library [38]. Index of the reference genome was built using Hisat2 v2.0.5 and paired-end clean 1 reads were aligned to the reference genome using Hisat2 v2.0.5 [39]. FeatureCounts [40] v1.5.0-p3 was used to count the reads numbers mapped to each gene. Then fragments per kilobase of transcript sequence per millions base pairs sequenced (FPKM) of each gene was calculated based on the length of the gene and reads count mapped to this gene. FPKM considers the effect of sequencing depth and gene length for the reads count at the same time and is currently the most commonly used method for estimating gene expression levels. Gene Ontology [41] (GO) enrichment analysis of differentially expressed genes was implemented by the clusterProfiler R package. GO terms with corrected P value less than 0.05 were considered significantly enriched by differential expressed genes. We used clusterProfiler R package to test the statistical enrichment of differential expression genes in KEGG [42] pathways. Gene Set Enrichment Analysis (GSEA) is a computational approach to determine if a pre- defined Gene Set can show a significant consistent difference between two biological states. The genes were ranked according to the degree of differential expression in the two samples, and then the predefined Gene Set were tested to see if they were enriched at the top or bottom of the list. A more detailed methodology can be found in the supplement.

### Western blotting

Following euthanasia, a lobe of the liver was frozen and crushed. Protein was extracted using RIPA lysis buffer and quantified using the Pierce Bicinchoninic Acid (BCA) Protein Assay Kit per manufacturer’s recommendation. 20 µg of protein was then loaded onto a precast Tris Glycine gel and electrophoresis was conducted at 100W. The protein was then transferred onto a PVDF membrane and blocked with 5% skim milk in 1 × TBST. The membrane was then incubated with fructose-1,6-bisphosphatase 1 (FBP1) primary antibody at a concentration of 1:1000 overnight at 4 °C followed by (anti-rabbit IgG) secondary antibody at a concentration of 1:5000. FBP1 bands were normalized to total protein as assessed by Ponceau staining.

### Hepatic triglyceride quantification

Hepatic triglyceride content was measured using a commercially available mouse Triglyceride ELISA kit (Abcam) according to the manufacturer’s protocol.

### Adipose and liver histology

Following euthanasia, a piece of pgWAT was fixed in 10% formalin and paraffin embedded and a fresh lobe of liver was frozen in optimal cutting temperature compound. Paraffin embedded and frozen samples were sliced into 5 µm sections and stained with Masson’s Trichome (pgWAT) or Oil-Red-O respectively, and an adjacent section was stained with Hematoxylin & Eosin (H&E) (Liver). Slides were scanned and assessed by an examiner blinded to the groups. To quantify pgWAT crown-like structures, images were opened in QuPath and 5 10 × 10 boxes (each individual box had a perimeter of 1000 µm and an area of 63,000µm^2^) were drawn for each sample. A blinded technician then counted the number of crown-like structures in each of the 5 boxes and took the average. Adipocyte size was quantified using AdipoSoft [43]. The 5 10 × 10 boxes drawn in QuPath were exported into Fiji and the AdipoSoft software was ran on each of the 5 10 × 10 boxes and the average of the 5 boxes was recorded. To quantify liver Oil-Red-O, images were opened in QuPath and 5 5 × 5 boxes were drawn per sample. Each box was then exported to Fiji. Green Channel images from an RGB stack were used for densitometric quantification of liver fat content. The average %Area of each box was taken and the average of the 5 boxes was recorded for each sample.

### Statistical analysis

Most group differences were determined by an unpaired parametric t-Test. In the T cell cytokine analysis, some values were below the assay limits of detection, therefore we used the non-parametric Mann–Whitney U test. Group differences in the glucose tolerance and insulin tolerance tests were assessed by repeated measures Two-Way ANOVA. For correlations, a simple linear regression was used to determine significant relations between body mass or pgWAT mass and the area under the curve (AUC) of the GTT. Data are reported as mean ± SEM with individual data points for most figures. On pie charts, data reported as mean only, data with individual data points along with mean ± SEM can be found in the supplement. For RNAseq data, Differential expression [44] analysis of two groups (two biological replicates per condition) was performed using the DESeq2Rpackage (1.20.0). DESeq2 provide statistical routines for determining differential expression in digital gene expression data using a model based on the negative binomial distribution.. Genes with an adjusted P value ≤ 0.05 found by DESeq2 were assigned as differentially expressed. (For edgeR [45] without biological replicates) Prior to differential gene expression analysis, for each sequenced library, the read counts were adjusted by edgeR program package through one scaling normalized factor. Differential expression analysis of two conditions was performed using the edgeR R package (3.22.5). The P values were adjusted using the Benjamini & Hochberg method. Corrected P value of 0.05 and absolute foldchange of 2 were set as the threshold for significantly differential expression.

## Results

### Early-life thymectomy results in greater body mass, frailty, and impaired glucose homeostasis

**Fig. 1:**
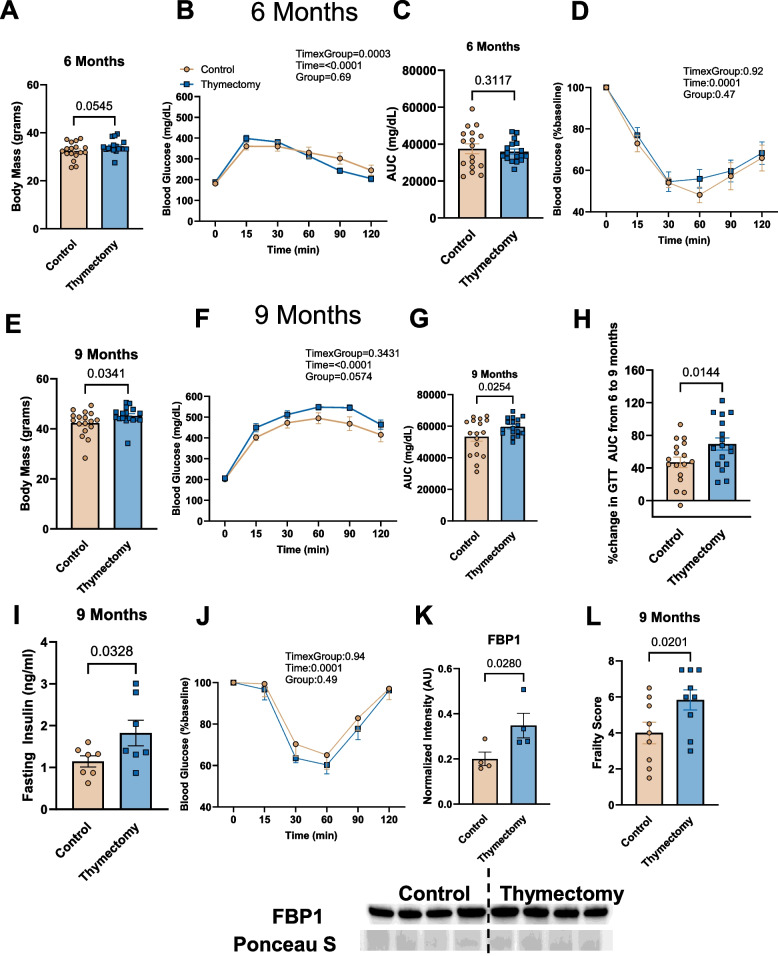
Early-life thymectomy results in greater body mass, glucose intolerance, and frailty. **A** Body mass of thymectomized and control mice at 6 months of age. **B** Blood glucose response curves during GTT in thymectomized and control mice at 6 months of age. **C** GTT area under the curve (AUC) in thymectomized and control mice at 6 months of age. **D** Blood glucose response curves during ITT in thymectomized and control mice at 6 months of age. **E** Body mass of thymectomized and control mice at 9 months of age. **F** Blood glucose response curves during GTT in thymectomized and control mice at 9 months of age. **G** GTT area under the curve in thymectomized and control mice at 9 months of age. **H** Percent increase in GTT AUC from 6 to 9 months in thymectomized and control mice. **I** Fasted at 9 months of age. **J** Blood glucose response curves during ITT in thymectomized and control mice at 9 months of age. **K** FBP1 protein expression in control and thymectomized mice. FPB1 was normalized to ponceau S stain. **L** 31-point frailty index score at 9 months of age. *N* = 4–17/group. Group differences in GTT and ITT were assessed by two-way repeated measures ANOVA. Other group differences were assessed by unpaired t-Test

At 6 months of age, thymectomized mice tended to have greater body mass compared to controls (*p* = 0.0545; Fig. 1A), but there were no differences in glucose or insulin tolerance between thymectomized and control mice at 6 months of age (Fig. 1B-D). At 9 months of age, thymectomized mice exhibited significantly greater body mass (Fig. 1E) and impaired glucose tolerance compared to controls (Fig. 1F & G). While both thymectomized and control mice exhibited increases in the GTT AUC from 6 to 9 months, the increase was exacerbated in thymectomized mice (Fig. 1H). Thymectomized mice also exhibited greater fasting insulins at 9 months of age (Fig. 1I). Thymectomized mice did not exhibit any differences in insulin sensitivity at 9 months of age compared to controls (Fig. 1J). We then assessed protein expression for FBP1, the rate limiting enzyme of gluconeogenesis. FBP1 protein content was greater in the liver of thymectomized mice compared to controls at 9 months of age (Fig. 1K), suggesting that thymectomized mice may exhibit greater gluconeogenesis. Finally, at 9 months of age, thymectomized mice exhibited greater frailty scores compared to their thymus intact counterparts (Fig. 1L), as assessed by a 31-point frailty index. This data demonstrates that early-life thymectomy is sufficient to induce greater body mass, impaired glucose tolerance and frailty, but does not induce peripheral insulin resistance.

**Fig. 2:**
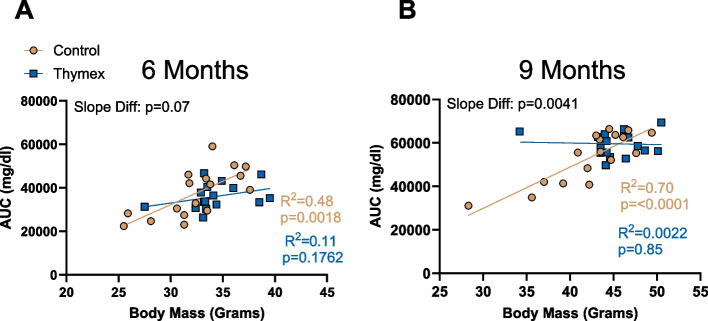
Early-life thymectomy uncouples the relation between body mass and glucose tolerance. **A** Relation between Body mass and GTT area under the curve at 6 months. **B** Relation between Body mass and GTT area under the curve at 9 months. *N* = 17/group. Group differences were assessed by simple linear regression

Greater body mass is associated with glucose intolerance in both mice and humans [46, 47]; therefore, we sought to determine if the impaired glucose tolerance in 9 month old thymectomized mice was related to their greater body mass. At 6 months of age, we found a significant and relatively strong correlation between body mass and AUC (*R*^2^ = 0.48, *p* = 0.0018; Fig. 2A) in control mice. However, thymectomized mice exhibited no significant relation between body mass and AUC (*R*^2^ = 0.11, *p* = 0.17; Fig. 2A) at 6 months of age. Importantly, at this time point, the difference between the slopes was not statistically significant. At 9 months of age, control mice demonstrated an even stronger relationship between body mass and AUC (*R*^2^ = 0.70, *p* = < 0.0001; Fig. 2B). In contrast the relation between body mass and AUC was completely uncoupled (*R*^2^ = 0.0022, *p* = 0.85; Fig. 2B) in 9 month old thymectomized mice. Furthermore, at this timepoint there was a significant difference between the two slopes (Fig. 2B). This data indicates that in young thymus intact mice, body mass and AUC are tightly related to one another, but this relationship becomes uncoupled in the thymectomized mice.

### Early-life thymectomy alters systemic T cell phenotype

**Fig. 3:**
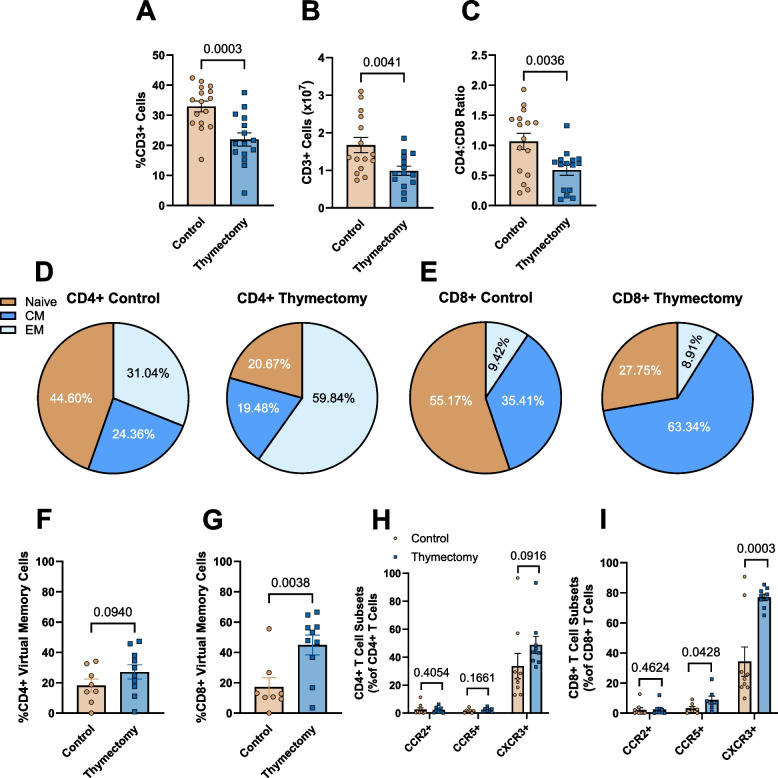
Early-life thymectomy results in an aged T cell phenotype in the spleen. Splenic single cell suspensions from control and thymectomized mice were stained with antibodies against CD3 (Pan T cell), CD4 (Helper T), CD8 (Cytotoxic T), CD44 (naïve/memory), CD62L (central/effector), CD49d (virtual memory), CCR2, CCR5, and CXCR3. T cell phenotype was assessed via flow cytometry. **A** Proportions (% of total leukocytes) of CD3 + T cells in the spleen. **B** Total number of CD3 + T cells in the spleen. **C** The CD4:CD8 ratio. **D** Proportions of CD4 + naïve, central memory (CM), and effector memory (EM) cells in the spleen. **E** Proportions of CD8 + naïve, CM, and EM cells in the spleen. **F** Proportions of CD4 + virtual memory cells. **G** Proportions of CD8 + virtual memory cells. **H** Proportions of CD4 + cells expressing chemokine receptors CCR2, CCR5, or CXCR3. **I** Proportions of CD8 + cells expressing chemokine receptors CCR2, CCR5, or CXCR3. *N* = 7–16/group. Data in pie charts expressed as mean only. Group Differences were assessed by unpaired t-Test

We next sought to determine the impact of thymectomy on T cell phenotype in the spleen at 9-months of age. There were no differences in the proportions or total number of CD45 + (Pan immune cells) in the spleen of thymectomized mice compared to controls (Supplemental Fig. 4A & B), however, thymectomized mice exhibited lower proportions and total number of pan (CD3 +) T cells compared to thymus intact controls (Fig. 3A & B). We also found that thymectomized mice exhibited a blunted splenic CD4:CD8 ratio, favoring CD8 + T cells (Fig. 3C). We next assessed naïve, central memory (CM), and effector memory (EM) phenotype, as well as virtual memory cells within the CD4 + and CD8 + T cell populations. In the CD4 + T cell pool, we found that thymectomized mice exhibited blunted proportions of naïve and greater proportions of EM, but not CM CD4 + T cells compared to thymus intact controls (Fig. 3D, Supplemental Fig. 4C). In the CD8 + T cell pool, thymectomized mice exhibited lower proportions of naïve and greater proportions of CM, but not EM CD8 + T cells (Fig. 3E, Supplemental Fig. 4D). We then assessed proportions of CD4 + and CD8 + virtual memory cells and found that thymectomized mice had greater proportions of CD8 +, but not CD4 + virtual memory cells in the spleen compared to controls (Fig. 3F & G). In thymectomized mice, T cell numbers are primarily maintained by homeostatic proliferation of existing T cells, a consequence of this proliferation is upregulation of the chemokine receptor CXCR3 [29]. To determine if thymectomy is associated with an increase in major T cell chemokine receptor expression, we assessed surface expression of CCR2, CCR5, and CXCR3 on the CD4 + and CD8 + T cells within the spleen. There were no differences in chemokine receptor expression within the CD4 + T cell pool (Fig. 3H). However, CD8 + T Cells from thymectomized mice exhibited significantly greater proportions of CD8 + CCR5 + and CD8 + CXCR3 + cells compared to controls (Fig. 3I). To determine whether one phenotype (e.g. memory/naïve) exhibited altered chemokine receptor expression in thymectomized mice, we assessed CCR2, CCR5, and CXCR3 on CD4 + and CD8 + naïve, central, and effector memory cells from the spleens of 9 month old control and thymectomized mice. There were no differences in the expression of CCR2, CCR5, or CXCR3 on any CD4 + T cell subtype (Supplemental Fig. 5A-C). In CD8 + T cells, naïve, central and effector memory cells from thymectomized mice all exhibited greater CXCR3 expression compared to controls (Supplemental Fig. 5D-F). Overall, these results indicate that thymectomy results in a systemic T cell phenotype that shares many features with chronological aging.

### T cells from thymectomized mice exhibit blunted proliferation, but greater cytokine production

**Fig. 4:**
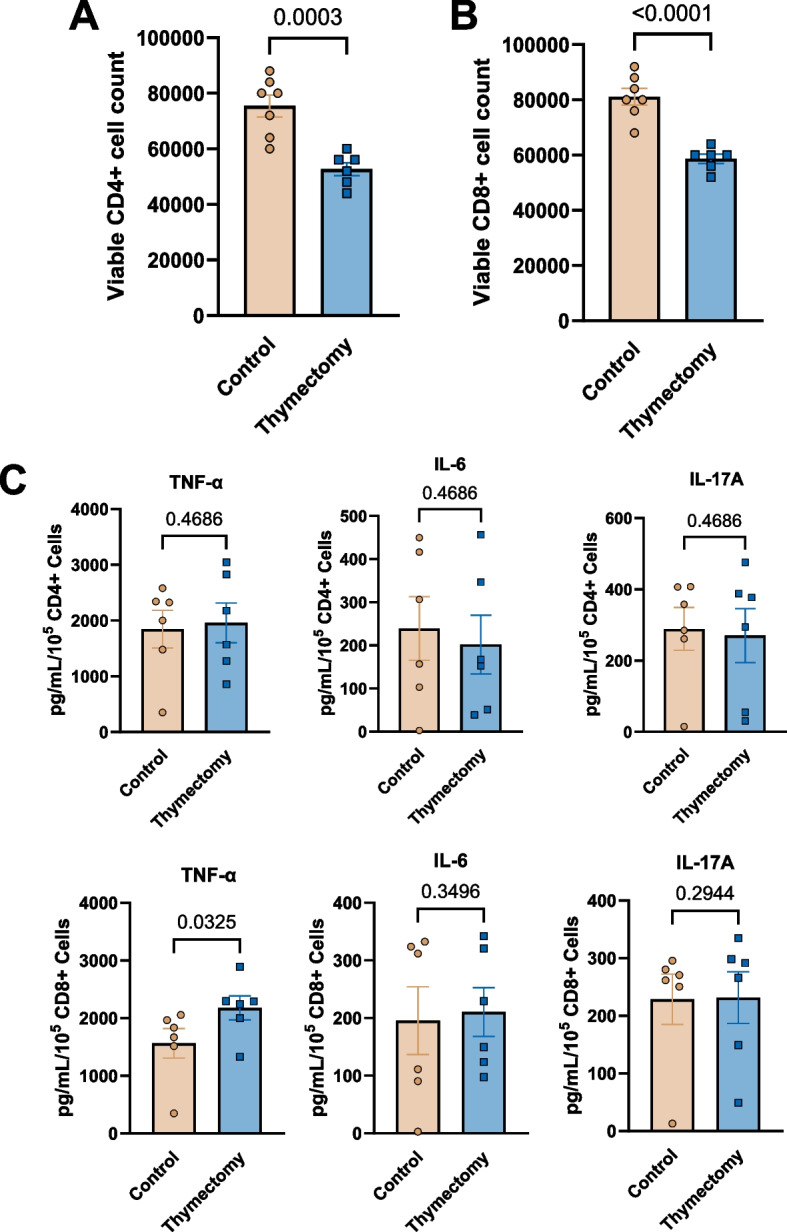
Splenic T cells from thymectomized mice exhibit blunted proliferation, but greater cytokine production. CD4 + and CD8 + T cells were isolated and stimulated with anti-CD3 and CD28 and allowed to proliferate for 96 h. A & B Number of CD4 + and CD8 + T cells following 96 h of proliferation. **C** Cytokine concentrations in the media following 96 h from CD4 + and CD8 + T cells normalized to 50,000 cells. *N* = 6–7/group. Group differences in panel (A & B) assessed by unpaired t-Test. Group differences in panel (**C**) assessed by Mann–Whitney U test

To assess T cell proliferation and cytokine production, 50,000 CD4 + and CD8 + T cells were isolated from the spleens of 9 month old thymectomized and control mice, activated with anti-CD3 and -CD28 beads and allowed to culture for 96 h and counted (separate wells for CD4 + and CD8 + cells). Both CD4 + and CD8 + cells from thymectomized mice exhibited blunted proliferation compared to their thymus intact counterparts (Fig. 4A & B). We then assessed cytokine concentrations in the cell culture media. As control mouse cells exhibited greater in vitro proliferation, we normalized cytokine concentrations per 50,000 cells. We found that CD8 + cells from thymectomized mice produced more tumor necrosis factor (TNF)-α compared to their thymus intact counterparts (Fig. 4C). Taken together, these data indicate that T cells in thymectomized mice exhibit blunted proliferation but are more inflammatory, largely consistent with T cell aging.

### Early-life thymectomy results in a memory, but not chemotactic T cell phenotype in the liver

**Fig. 5:**
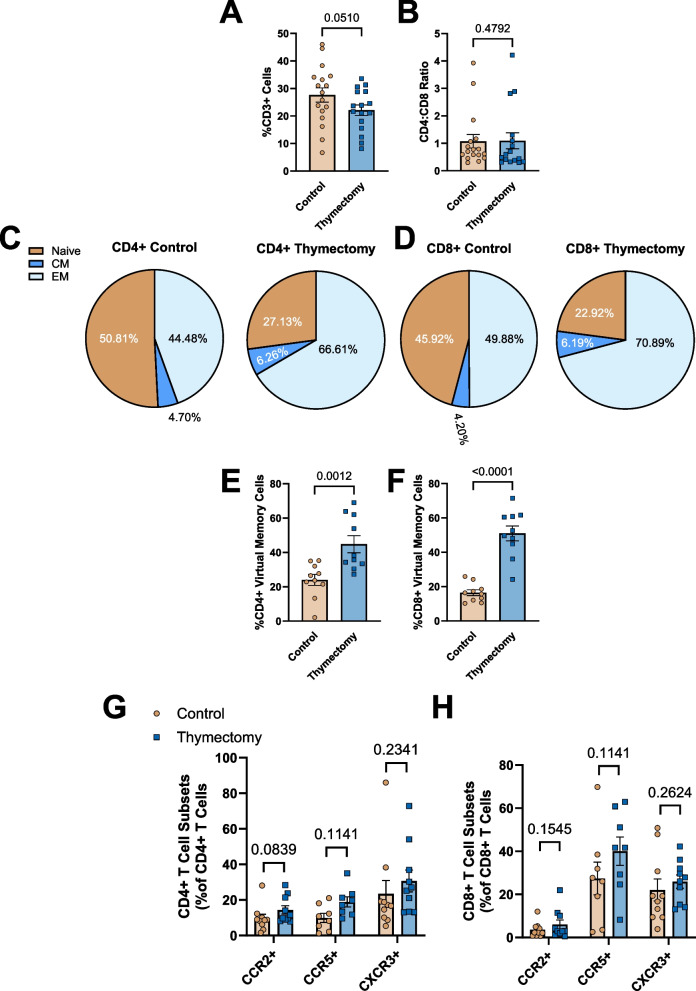
T cells in the livers of thymectomized mice exhibit an aging phenotype. Liver single cell suspensions from control and thymectomized mice were stained with antibodies against CD3 (Pan T cell), CD4 (Helper T), CD8 (Cytotoxic T), CD44 (naïve/memory), CD62L (central/effector), CD49d (virtual memory), CCR2, CCR5, and CXCR3. T cell phenotype was assessed via flow cytometry. **A** Proportions (percent of total leukocytes) CD3 + T cells. **B** The CD4:CD8 ratio. **C** Proportions of CD4 + naïve, central memory (CM), and effector memory (EM) cells in the liver. **D** Proportions of CD8 + naïve, CM, and EM cells in the liver. **E** Proportions of CD4 + virtual memory cells. **F** Proportions of CD8 + virtual memory cells. **G** Proportions of CD4 + cells expressing chemokine receptors CCR2, CCR5, or CXCR3. **H** Proportions of CD8 + cells expressing chemokine receptors CCR2, CCR5, or CXCR3. *N* = 7 = 17/group. Group Differences assessed by unpaired t-Test. Data in pie charts expressed as mean only

We and others have previously established that T cells accumulate in the livers of old mice and directly contribute to age-related liver inflammation and hepatic function [7, 8]. Using flow cytometry, we quantified total immune and T cell populations within the liver of thymectomized and control mice at 9 months of age. There were no differences in the proportion or total number per gram of CD45 + cells in the livers of thymectomized and control mice (Supplemental Fig. 6A & B). Thymectomized mice exhibited blunted proportions of CD3 + T cells, but no differences in the total number of CD3 + T cells per gram of liver (Fig. 5A; Supplemental Fig. 6C). There were no differences in the CD4:CD8 ratio between thymectomized and control mice (Fig. 5B). We then assessed the naïve and memory phenotype of CD4 + and CD8 + cells in thymectomized mice. In the CD4 + T cell pool, thymectomized mice exhibited significantly blunted proportions of naïve and significantly greater proportions of EM, but not CM CD4 + T cells compared to controls (Fig. 5C; Supplement Fig. 6D). Similarly, within the CD8 + T cell pool, thymectomized mice exhibited significantly blunted proportions of CD8 + naïve T cells, and greater proportions of EM, but not CM CD8 + T cells compared to controls (Fig. 5D, Supplement Fig. 6E). Finally, we assessed hepatic CD4 + and CD8 + virtual memory cells. Thymectomized mice exhibited significantly greater proportions of both CD4 + and CD8 + virtual memory cells (Fig. 5E & F). These data suggest that thymectomy induced alterations that occur in the lymphoid organs also occur in other tissues.

We next sought to determine if hepatic T cells exhibited greater chemokine receptor expression in thymectomized mice. Within the CD4 + T cell pool there were no differences in the proportions of CCR2 +, CCR5 + or CXCR3 + CD4 + T cells between thymectomized and control mice (Fig. 5G). Similarly, within the CD8 + T cell pool, there were no differences in the proportions of CCR2 +, CCR5 +, or CXCR3 + CD8 + T cells between thymectomized and control mice (Fig. 5H). CD4 + naïve cells from thymectomized mice exhibited greater expression of CCR2 and CCR5, but not CXCR3 compared to controls (Supplemental Fig. 7A). There were no differences in chemokine expression in any other subtypes (Supplemental Fig. 7B-F)

### Early life thymectomy results in an aged and chemotactic T cell phenotype in the pgWAT

**Fig. 6:**
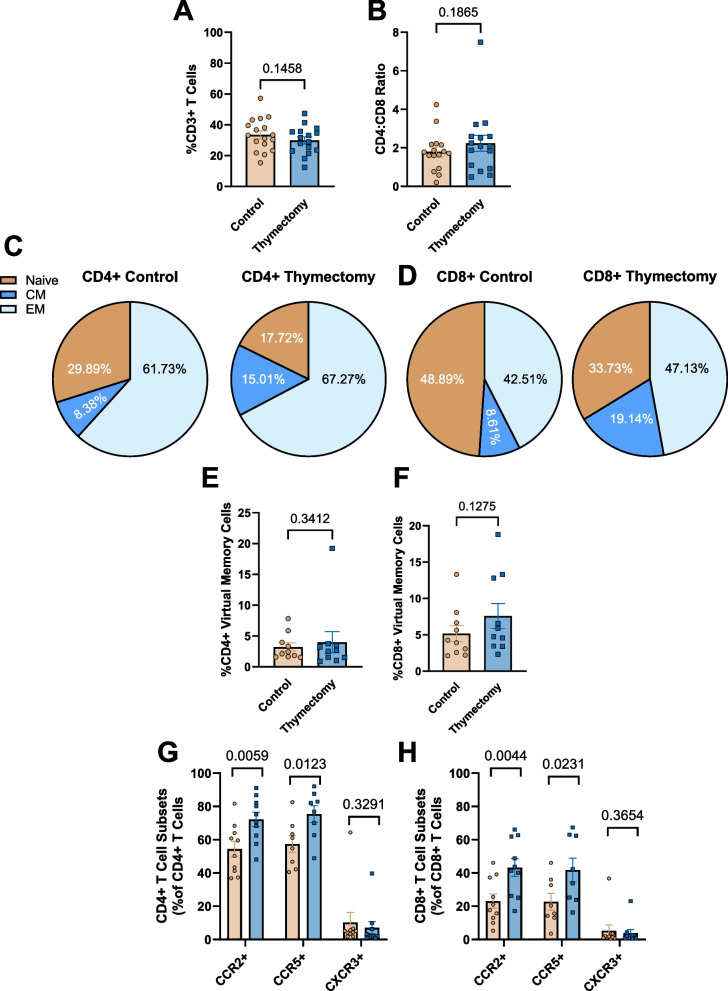
The pgWAT of thymectomized mice exhibit a T cell phenotype that is similar to aging. pgWAT single cell suspensions from control and thymectomized mice were stained with antibodies against CD3 (Pan T cell), CD4 (Helper T), CD8 (Cytotoxic T), CD44 (naïve/memory), CD62L (central/effector), CD49d (virtual memory), CCR2, CCR5, and CXCR3. T cell phenotype was assessed via flow cytometry. **A** Proportions (percent of total leukocytes) CD3 + T cells. **B** The CD4:CD8 ratio. **C** Proportions of CD4 + naïve, central memory (CM), and effector memory (EM) cells in the pgWAT. **D** Proportions of CD8 + naïve, CM, and EM cells in the pgWAT. **E** Proportions of CD4 + virtual memory cells. **F** Proportions of CD8 + virtual memory cells. **G** Proportions of CD4 + cells expressing chemokine receptors CCR2, CCR5, or CXCR3. **H** Proportions of CD8 + cells expressing chemokine receptors CCR2, CCR5, or CXCR3. *N* = 7–17/group. Group Differences assessed by unpaired t-Test. Data in pie charts expressed as mean only

We next sought to determine the T cell phenotype within the pgWAT of thymectomized and control mice at 9 months. There were no differences in the proportions or total number of CD45 + cells per gram of pgWAT in thymectomized and control mice (Supplement Fig. 8A & B). There were also no differences in the proportions or total number per gram of CD3 + T cells in the pgWAT (Fig. 6A; Supplemental Fig. 8C), along with no differences in the CD4:CD8 ratio (Fig. 6B). We then assessed T cell naïve and memory phenotype of the pgWAT in thymectomized and control mice. In the CD4 + T cell pool, thymectomized mice exhibited significantly lower proportions of CD4 + naïve cells compared to controls that was not explained by an expansion of either CM or EM cells alone (Fig. 6C, supplementary Fig. 8D). In the CD8 + T cell pool, there were no alterations in the proportion of CD8 + naïve cells between thymectomized mice and controls (Fig. 6D, Supplementary Fig. 8E). Thymectomized mice exhibited significantly greater proportions of CD8 + CM, but not EM cells compared to controls (Fig. 6D, Supplementary Fig. 8E). There were also no differences in the proportions of CD4 + or CD8 + virtual memory cells in the pgWAT of thymectomized and control mice (Fig. 6E & F). We next assessed CD4 + and CD8 + T cell chemokine receptor expression in T cells of the pgWAT. Within the CD4 + T cell pool, thymectomized mice exhibited greater proportions of CD4 + CCR2 + and CD4 + CCR5 + cells compared to controls with no difference in the proportions of CD4 + CXCR3 + cells (Fig. 6G). Within the CD8 + T cell pool, thymectomized mice exhibited greater proportions of CD8 + CCR2 + and CD8 + CCR5 + cells with no differences in the proportions of CD8 + CXCR3 + cells compared to controls (Fig. 6H). We then assessed surface expression of CCR2, CCR5, and CXCR3 on CD4 + and CD8 + naïve, central, and effector memory cells from the pgWAT of control and thymectomized mice at 9 months. CCR2 was higher in all 3 subtypes and CCR5 was primarily elevated in naïve cells (Supplemental Fig. 9A-C). CD8 + cells exhibited greater CCR2 and CCR5 expression, but this was not explained by elevation in a specific subtype alone (Supplemental Fig. 9D-F). Collectively, these data indicate that after thymectomy there is a shift towards a memory T cell phenotype in the pgWAT. Further, while the greater chemokine expression is not explained by any single subtype, the presence of greater overall proportions of CCR2 + and CCR5 + T cells suggest that these cells are being recruited to the pgWAT.

### Bulk RNASeq of the liver and visceral adipose tissue reveal a dysregulated immune system and altered metabolic gene expression

Because we observed an accumulation of memory T cells in both the pgWAT and liver we employed bulk RNAseq to determine whether T cell phenotype could induce transcriptional changes on the whole tissue level. Initial principal component analysis revealed separation between the pgWAT of thymectomized and control mice, whereas the livers from these two groups were clustered closely together (Supplemental Fig. 10A). Likewise, the pgWAT had a total of 5324 differentially expressed genes (DEG) in thymectomized mice, whereas the liver had 1058 DEG (Supplemental Fig. 10B).

We first examined DEG in the liver of thymectomized and control mice. In thymectomized mice, we observed an upregulation of novel genes that have demonstrated to either be causal (*Mogat1, Pklr*) or associated with liver steatosis (*Serpina7,* and *Hcn3*). *Pck1*, a gene that may protect against hepatic steatosis was downregulated in thymectomized mice (Supplemental Fig. 10C & 11A). We then preformed Gene Ontology (GO) analysis using the DEG of the liver. The liver of thymectomized mice did not exhibit terms that were associated with the immune response and inflammation, rather they exhibited greater upregulation of genes responsible organic acid synthesis and generation of precursor metabolites and energy (Supplemental Fig. 11B & C).

**Fig. 7:**
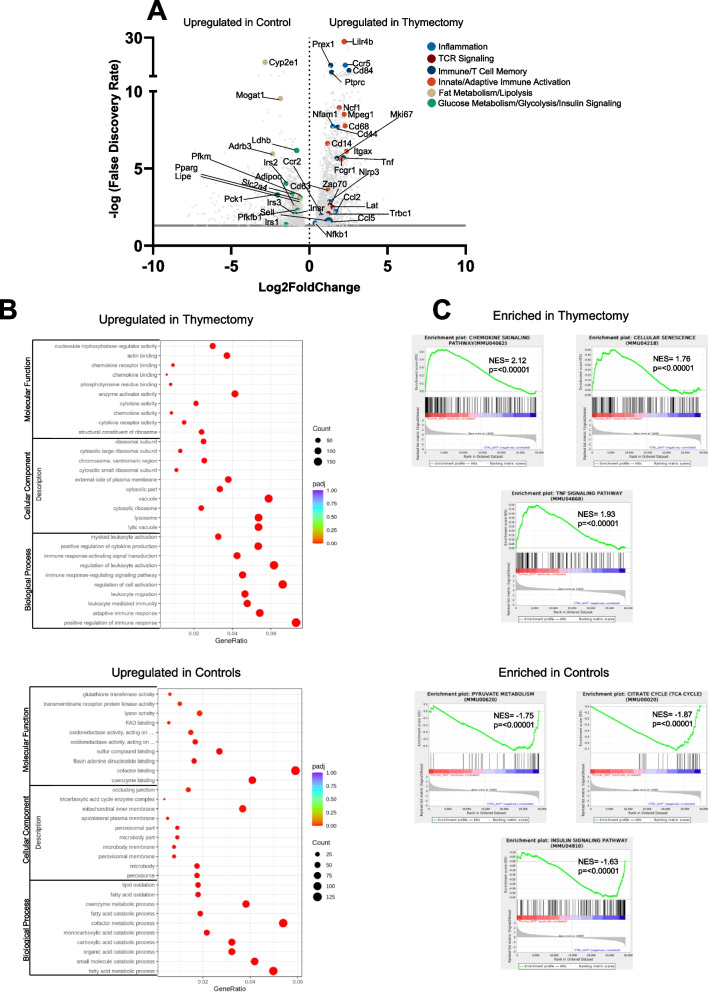
pgWAT from thymectomized mice exhibits inflammatory immune mediated gene expression. **A** Volcano plot showing only significantly upregulated DEG after adjusting for FDR from the pgWAT in thymectomized and control mice. **B** GO terms that are upregulated in pgWAT of thymectomized or control animals. **C** GSEA analysis of KEGG pathways that are enriched within the pgWAT in control or thymectomized mice. *N* = 4/group. The P values were adjusted using the Benjamini & Hochberg method. GSEA analysis report normalized enrichment score and nominal p value

We next examined DEG in the pgWAT of thymectomized and control mice. Thymectomized mice exhibited downregulation of various genes responsible for lipolysis (*Cyp2e1*, *Mogat1*, *Adrb3*, *Adipoq, Pparg,* and *Lipe),* insulin signaling (*Irs1*, *Irs2*, *Irs3*, and *Insr),* and carbohydrate metabolism (*Pfkfb, Pfkm, Pck1, Ldhb,* and *Slc2a4).* Whereas thymectomized mice exhibited an upregulation of genes responsible for 1) Inflammation (*Ccr5, Ccr2, Nfam1*, *Ccl2*, *Ccl5*, *Nfkb1*, and *Nlrp3*), 2) TCR Signaling (*Zap70*, *Trbc1*, and *Lat*), 3) Immune/T cell memory (*Prex1*, *Cd84*, *Cd44*, *Mki67*, *Tnf*, and *Sell*), and 4) Innate/Adaptive immune activation (*Lilr4b*, *Ncf1*, *Mpeg1*, *Cd68*, *Cd14*, *Fcgr1*, and *Cd63*) (Fig. 7A, Supplemental Fig. 10D).

We then performed gene ontology analysis (GO) in DEG from the pgWAT. GO terms related to immune response, activation, migration, and cytokine production were significantly upregulated in thymectomized mice (Fig. 7B). Whereas GO terms related to oxidation and anti-inflammatory responses and metabolic function were upregulated in control mice (Fig. 7B). We then performed GSEA analysis on KEGG pathways generated from DEG within the pgWAT. Genes in pathways such as chemokine and TNF signaling as well as cellular senescence were significantly enriched in thymectomized mice, whereas genes in pathways responsible for pyruvate metabolism, Citrate (TCA) cycle, and insulin signaling were enriched in controls (Fig. 5C). These data suggest that thymectomy results in pgWAT inflammation (*Nfkb1*/*Nlrp3*), as well as activation and recruitment of both the innate (*Cd14*/*Fcgr1*) and adaptive immune systems (*Cd44*/*Zap70*) and subsequent immune cell recruitment (*Ccr5/Ccl5*) to the pgWAT.

### Early-life thymectomy results in greater hepatic lipid deposition and histopathological inflammation of the pgWAT

**Fig. 8:**
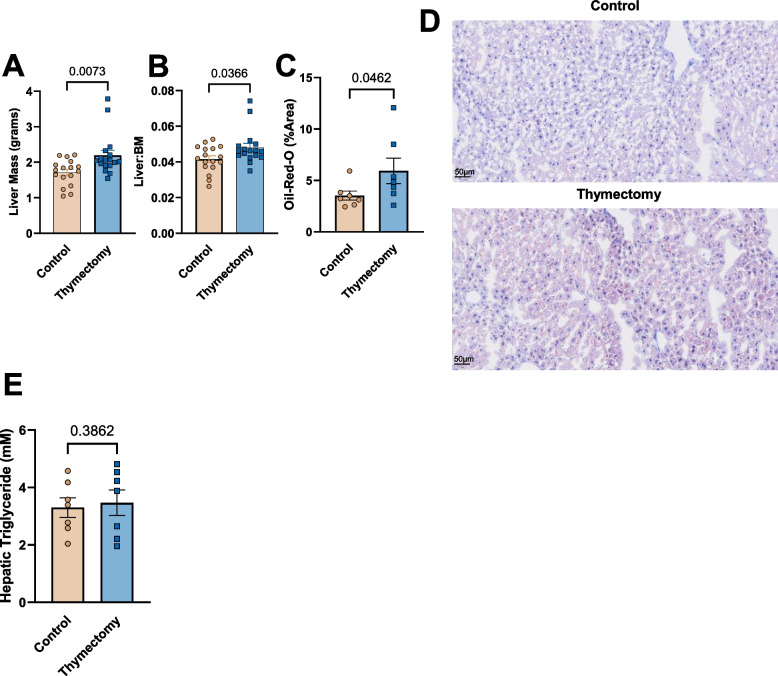
Early-life thymectomy results in hepatic lipid deposition (**A**) Liver mass at 9 months of age. **B** Liver:body mass ratio, a marker of hepatic steatosis. **C** Livers from thymectomized and control mice were stained with Oil-Red-O and %area was quantified using imageJ. **D** Representative images of Oil-Red-O. **E** Hepatic Triglyceride content. *N* = 4–17/group. Group differences were assessed by unpaired t-Test

We assessed liver mass, the liver:Body-mass ratio, hepatic lipid content using oil-red-o, and quantified hepatic triglyceride content at 9 months. Thymectomized mice exhibited greater liver mass as well as a greater liver:BM ratio (Fig. 8A & B). Thymectomized mice also exhibited greater hepatic lipid content compared to controls (Fig. 8C & D), however there were no differences in hepatic triglyceride content (Fig. 8E).

**Fig. 9:**
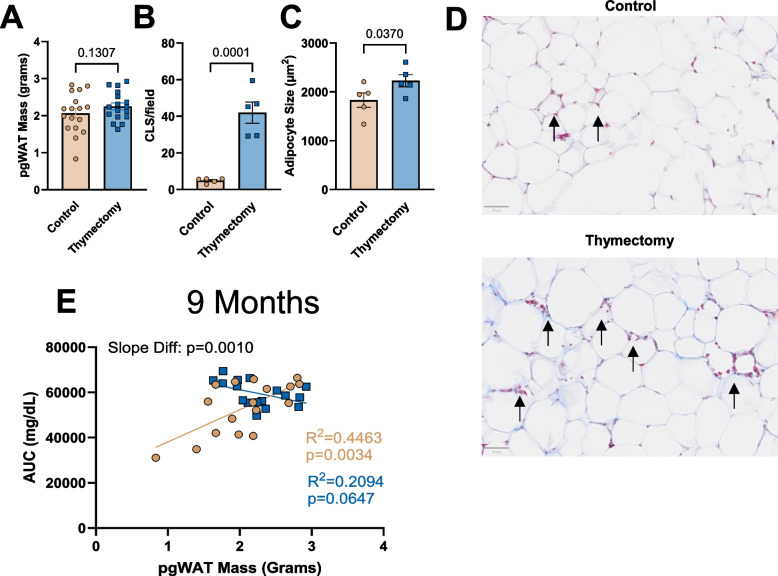
Early-life thymectomy results in histopathological inflammation of the pgWAT. **A** pgWAT mass at 9 months of age. **B** Section of pgWAT were stained with masons trichrome and crown-like structures were quantified. **C** Quantification of Adipocyte size. **D** Representative images Masson’s trichrome images of pgWAT with arrows pointing at crown-like structures. **E** Relation between pgWAT mass and GTT area under the curve. *N* = 5–17/group. Group differences were assessed by unpaired t-Test or simple linear regression

We next assessed pgWAT fat pad mass and found no differences between thymectomized and control mice (Fig. 9A). We then assessed crown-like structures, a histopathological marker of inflammation, and adipocyte size in control and thymectomized mice. Thymectomized mice exhibited greater crown-like structures and average adipocyte size compared to control mice (Fig. 9B-D). In obese animals, GTT AUC has been reported to significantly correlate with pgWAT mass [46]. Therefore, we correlated the GTT AUC with 9 month pgWAT mass in control and thymectomized animals. Control mice exhibited a moderately strong and significant relation between GTT AUC and pgWAT mass (*R*^2^ = 0.4463, *p* = 0.0034; Fig. 9E). Whereas thymectomized mice exhibited a relatively weak and non-significant relation between GTT AUC and pgWAT mass (*R*^2^ = 0.2094, *p* = 0.0647; Fig. 9E). Importantly, there was a significant difference between the slopes, suggesting that the uncoupling between body mass and GTT AUC (Fig. 2) and pgWAT mass and GTT AUC (Fig. 9E) in thymectomized mice is being driven by T cell mediated immune dysregulation and subsequent inflammation of the pgWAT even in the absence of pgWAT adipose tissue expansion.

## Discussion

The principal findings of this investigation are as follows: 1) Early life thymectomy induces greater body mass, impaired glucose tolerance, and frailty at 9 months of age. 2) Early life thymectomy results in a shift in T from a naïve towards a memory cell phenotype in the spleen, liver, and pgWAT. 4) The pgWAT of thymectomized mice exhibits a more pro-inflammatory phenotype in the absence of greater overall T cell accumulation or adipose tissue expansion. Taken together, these observations suggest that early life thymectomy is sufficient to induce glucose intolerance and visceral adipose tissue inflammation in 9 month old adult mice.

In the present study we observed that early life thymectomy results in greater body mass and impaired glucose tolerance, but not peripheral insulin resistance at 9 months of age. Although there is a previous report demonstrating that early-life thymectomy induces glucose intolerance in rats [48], this previous study did not implicate T cells per se as the underlying mediator of glucose intolerance, as the function of the thymus was not yet fully established [49]. We initially evaluated body mass, glucose, and insulin tolerance at 6 months of age and although thymectomized mice tended to weigh more, there was no impairment in glucose or insulin tolerance. However, at 9-months of age, thymectomized mice exhibited greater overall body mass, glucose intolerance, and greater fasting insulin. We did not observe differences in insulin sensitivity. Our observation of glucose intolerance in the absence of altered insulin sensitivity suggests that one or both of the following may have occurred in our thymectomized mice: 1) That liver and/or the pgWAT, but not the skeletal muscle was insulin resistant, but the ITT was not sensitive enough to detect these differences in insulin sensitivity (*i.e.* only 10–20% insulin-stimulated glucose uptake in liver and adipose, 80% in skeletal muscle [50]). 2) That thymectomized mice exhibited greater liver gluconeogenesis leading to impaired glucose tolerance at 9 months. Our finding that thymectomized mice exhibit greater FBP1 protein expression compared to controls supports the latter concept. It should be noted that although our 9 month old control mice exhibited relatively high body weights, these mice were still well within normal established body mass ranges for their age [51].

We are the first to observe that early life thymectomy results in greater frailty in adult mice. There is some evidence linking impaired glucose tolerance to frailty in humans [5]. Complementing our observations, induction of pre-mature thymic atrophy or induction of DNA damage in all immune cells results in senescence in solid organs and premature death but no overt differences in body weight [14, 52]. Induction of mitochondrial dysfunction in CD4 + T cells induces some features of frailty, substantial weight loss, and premature death [13]. Our observation that thymectomized mice weighed more than controls suggest that each of these models of premature immune aging do not act in an entirely uniform manner; however, collectively these studies underscore the concept that the immune system can drive aging and dysfunction of other tissues.

We and others have employed early life thymectomy as a model of T cell aging [24–29]. When the thymus is removed, mice are unable to generate new T cells in the thymus and are therefore reliant upon proliferation of existing T cells to maintain the T cell pool [53]. T cells undergo many changes during aging including: a blunted CD4:CD8 ratio, decreased proportions of naïve and increased proportions of memory T cells, increased proportions of virtual memory T cells, as well as a proinflammatory phenotype [7, 14, 17, 24, 25, 35–37, 54–59]. In this investigation, we demonstrated that the CD4:CD8 ratio was blunted in the spleen, which is consistent with our previous observations [7, 24]. We and others have also previously observed that T cells in thymectomized mice exhibited blunted proportions of naïve and greater proportions of memory cells [24, 25, 29], however, most of these observations were in the spleen or other lymphoid organs [25, 29]. We have recently observed a similar shift towards a memory phenotype with thymectomy in T cells accumulating around the arteries [24] and in the current investigation extend these observations to the liver and pgWAT. The blunted proliferative capacity of both CD4 + and CD8 + T cells, but greater cytokine production in CD8 + T cells is in agreement with previous observations [29]. Thymectomized mice had greater proportions of CD8 + virtual memory cells in the spleen and liver, but not the pgWAT. These observations are consistent with our observations in the blood, aorta, and mesenteric adipose tissue of thymectomized mice [24]. Virtual memory cells exhibit blunted proliferation and a greater proinflammatory phenotype which is a similar phenotype to what we observed in our thymectomized mice [36]. Together, these data suggest that thymectomy induces many phenotypic changes that are similar to those observed with chronological aging; however, the precise subtype of immune cells that contributes to dysfunction of other organs in thymectomized animals is not yet understood.

Thymectomy results in greater splenic CXCR3 + CD8 + cells that produce TNF-α [29]. We examined major T cell chemokine receptor expression and also found that a greater proportion of splenic CD8 + cells from thymectomized mice were CXCR3 +. We also observed a modest elevation in the proportion of splenic CCR5 + CD8 + T cells in thymectomized mice. In liver T cells, thymectomy did not alter chemokine receptor expression. In contrast, in the pgWAT thymectomy resulted in greater CCR2 + and CCR5 + expression on both CD4 + and CD8 + cells. In addition, the proportion of CCR2 + and CCR5 + cells were relatively low in the spleen of both the thymectomized and control mice (< 10%) but substantially greater in the pgWAT (> 40%). Our pgWAT RNAseq data also suggests an upregulation of immune cell trafficking. Notably, GO terms related to chemokine activity were highly upregulated with thymectomy and *Ccr5* was one of the top 10 upregulated transcripts. These data suggest that thymectomy alters T cell chemokine receptor expression and CCR2 and CCR5 mediated inflammation of the pgWAT.

The “portal hypothesis” is the concept that liver is the first organ exposed to molecules from the visceral adipose via the portal vein which can cause hepatic inflammation and dysfunction [60]. While we did not observe substantial inflammation in the livers of thymectomized mice. We did observe greater overall hepatic lipid accumulation as well as some evidence of gene expression changes in the liver that would be consistent with greater lipid accumulation. For example, *Mogat1 and Pklr,* were both upregulated in the liver of thymectomized mice play a role in liver metabolic dysfunction[61–63]. Whereas *Pck1*, which was downregulated in thymectomized mice has been demonstrated to be protective of diet induced hepatic steatosis [64]. Thymectomy also resulted in upregulation of *Serpina7* [65] and *Hcn3* [66] which are highly upregulated in various models and tissues of diet induced steatosis, however the exact role of these genes in hepatic dysfunction is still unclear. Despite some of these changes in gene expression and overall hepatic lipid accumulation, we did not observe greater hepatic triglyceride in thymectomized mice. However, our data demonstrating greater FBP1 protein and greater overall hepatic lipid deposition (oil-red-o) supports the portal hypothesis as a potential mechanism of overall hepatic dysfunction.

The pgWAT of thymectomized mice demonstrated the most substantial changes in gene expression. A majority of downregulated genes observed in the pgWAT with thymectomy were those related to metabolic function, in particular genes related to fatty acid metabolism. Our observations also suggest that thymectomy results in an inappropriate activation and mobilization of the immune system within the pgWAT that leads to tissue inflammation and ultimately physiologic decline. These data also indicate that pgWAT inflammation is not exclusively T cell mediated as GO terms related to myeloid leukocyte activation and adaptive immune response were upregulated with thymectomy. Thymectomized mice exhibited a greater number of adipose crown like structures, also supporting the role of multiple immune cell subtypes in pgWAT inflammation. Further, our observation that glucose tolerance was uncoupled from body and adipose mass in the thymectomized mice suggests that visceral adipose inflammation may contribute to worsened glucose tolerance observed in these mice. Collectively these results suggest that T cells from thymectomized mice may orchestrate inflammation of the pgWAT that involves multiple immune cell types and results in glucose intolerance.

Our data also suggests that thymectomy may also induce cellular senescence within the pgWAT. Senescent cells accumulate within the adipose tissue and are associated with inflammation in humans [67]. Further, it has been demonstrated that genetic clearance of senescent cells or senolytic treatment results in blunted pgWAT senescence and inflammation and improved glucose tolerance and frailty in old mice [8, 68, 69]. We observed an enrichment of genes associated with cellular senescence in the pgWAT of thymectomized mice. Similarly, a recent investigation analyzed single cell RNAseq of cell types that expressed high levels of the senescence gene, *p21* in the pgWAT of high fat fed mice. They found an upregulation of genes that play a role in the immune response, chemotaxis, chemokine signaling, and TNF signaling pathway [70]. Notably, these genesets were also upregulated in our thymectomized mice. However, *Cdkn1a*, the p21 gene, was not significantly upregulated (after adjusting for FDR) in thymectomized mice, it is possible that bulk RNAseq is not sensitive enough to distinguish between upregulation of inflammation associated genes and senescence associated genes. For example, many (*Nfkb1/Il6*) but not all (*p53/Foxo33*) of the senescence associated upregulated genes we observed are also related to inflammatory processes. 

There are some limitations to the present study. The purpose of this study was to determine whether early life thymectomy would result in an alteration of T cell phenotype in metabolically active tissues as well as result in impaired glucose tolerance. While we tested this initial hypothesis, the precise mechanism of impaired glucose tolerance in thymectomized mice is unclear. Whether food intake and/or energy expenditure are altered, and which tissue is specifically responsible for impaired glucose tolerance with thymectomy remain unknown. The altered proportions of CD45 + cells, histology, and RNA-seq data from the pgWAT suggest that other immune cells may play a role in adipose tissue inflammation. Because our hypothesis was to determine whether thymectomy affected T cell phenotype, we did not a priori, set out to perform a comprehensive immunophenotyping of other immune cells. Lastly, in humans, thymic involution begins at sexual maturity whereas in mice it occurs relatively later in life (~ 18 months). Thus, the direct application of our data to humans is unknown. Further, whether T cell aging and/or thymectomy in humans result in similar adipose inflammation and glucose intolerance as observed here is unknown.

## Conclusion

In conclusion, we demonstrate that early life thymectomy results in glucose intolerance that is independent of increases in body mass and adipose tissue expansion, along with increased frailty at 9 months of age. In addition, thymectomized mice exhibit changes in T cell phenotype that are consistent with aging. These results support the concept that aged T cells alone can contribute to metabolic impairments and adipose tissue dysfunction, even in adult animals. Further, our results may have clinical implications for children who undergo cardiac surgery and associated thymectomy at a young age.

## Supplementary Information

Supplementary Material 1.

## Data Availability

The datasets used and/or analyzed during the current study are available from the corresponding author on reasonable request.

## Abbreviations

pgWAT: Perigonadal Adipose Tissue
CD45: Pan Immune Cells
CD3: Pan T Cells
CD4: Helper T Cells
CD8: Cytotoxic T Cells
CM: Central Memory T Cell
EM: Effector Memory T Cell
BCA: Bicinchoninic acid
FBP1: Fructose-1,6-bisphosphatase 1
DEG: Differentially Expressed Gene
GO: Gene Ontology
KEGG: Kyoto Encyclopedia of Genes and Genomes
GSEA: Gene Set Enrichment Analysis
BM: Body Mass
GTT: Glucose Tolerance Test
ITT: Insulin Tolerance Test
AUC: Area Under the Curve
TNF-α: Tumor Necrosis Factor-α
TNF: Tumor Necrosis Factor
