# Supplementary material for: Early-life thymectomy results in visceral adipose tissue inflammation and glucose intolerance
Source: Immun Ageing. 2025 Oct 1;22:36. doi: 10.1186/s12979-025-00531-x (PMC12487299; doi:10.1186/s12979-025-00531-x)
Supplement: Supplementary file 1 — Supplementary Material 1. [file 12979_2025_531_MOESM1_ESM.zip › Buckley et al supplemental material/Thymectomy Supplemental Figure Legends Revision.pdf]

Supplemental Figure 1: T cell gating strategy in the spleen. Spleens from thymectomized and control mice were dissected, mechanically disrupted and stained with anti-CD45 (leukocytes), anti-CD3 (pan T cells), anti-CD4 (Helper T), anti-CD8 (Cytotoxic T), anti-CD44 (naïve/memory), anti-CD62L (central/effector), anti-CD49d (Virtual memory), anti-CCR2, anti-CCR5, anti-CXCR3, and violetFluor 450 Ghost dye (exclusion of dead cells).

Supplemental Figure 2: T cell gating strategy in the Liver. Livers from thymectomized and control mice were dissected, mechanically disrupted, enzymatically digested, and stained with anti-CD45 (leukocytes), anti-CD3 (pan T cells), anti-CD4 (Helper T), anti-CD8 (Cytotoxic T), anti-CD44 (naïve/memory), anti-CD62L (central/effector), anti-CD49d (Virtual memory), anti-CCR2, anti-CCR5, anti-CXCR3, and violetFluor 450 Ghost dye (exclusion of dead cells).

Supplemental Figure 3: T cell gating strategy in the pgWAT. pgWAT from thymectomized and control mice were dissected, mechanically disrupted, enzymatically digested, and stained with anti-CD45 (leukocytes), anti-CD3 (pan T cells), anti-CD4 (Helper T), anti-CD8 (Cytotoxic T), anti-CD44 (naïve/memory), anti-CD62L (central/effector), anti-CD49d (Virtual memory), anti-CCR2, anti-CCR5, anti-CXCR3, and violetFluor 450 Ghost dye (exclusion of dead cells).

Supplemental Figure 4: T cell phenotype in the Spleen. Spleens from thymectomized and control mice were dissected, mechanically disrupted, enzymatically digested, and stained with anti-CD45 (leukocytes), anti-CD3 (pan T cells), anti-CD4 (Helper T), anti-CD8 (Cytotoxic T), anti-CD44 (naïve/memory), and anti-CD62L (central/effector). **(A)** proportion of leukocytes in the spleen. **(B)** Total number of

Leukocytes in the spleen. **(C)** Proportions of CD4+ Naïve, central memory (CM), and effector memory (EM) cells. **(D)** Proportions of CD8+ Naïve, central memory (CM), and effector memory (EM) cells. N=12-16/group. Group differences assessed by unpaired t-Test.

Supplemental Figure 5: T cell phenotype and chemokine receptor in the Spleen. Spleens from thymectomized and control mice were dissected, mechanically disrupted and stained with anti-CD45 (leukocytes), anti-CD3 (pan T cells), anti-CD4 (Helper T), anti-CD8 (Cytotoxic T), anti-CD44 (naïve/memory), and anti-CD62L (central/effector), anti-CCR2, anit-CCR5, and anti-CXCR3. **(A-C)** CD4+ T cell phenotype (naïve, central memory, and effector memory) and chemokine receptor expression in the spleen. **(D-F)** CD8+ T cell phenotype (naïve, central memory, and effector memory) and chemokine receptor expression in the spleen. N=8-10/group. Group differences assessed by unpaired t-test.

Supplemental Figure 6: T cell phenotype in the liver. Livers from thymectomized and control mice were dissected, mechanically disrupted, enzymatically digested, and stained with anti-CD45 (leukocytes), anti-CD3 (pan T cells), anti-CD4 (Helper T), anti-CD8 (Cytotoxic T), anti-CD44 (naïve/memory), and anti-CD62L (central/effector). **(A)** proportion of leukocytes in the liver. **(B)** Total number of Leukocytes per gram of tissue in the liver. **(C)** Total number of CD3+ T cells per gram of tissue in the liver. **(D)** Proportions of CD4+ Naïve, central memory (CM), and effector memory (EM) cells. **(E)** Proportions of CD8+ Naïve, central memory (CM), and effector memory (EM) cells. N=13-17/group. Group differences assessed by unpaired t-Test.

Supplemental Figure 7: T cell phenotype and chemokine receptor in the liver. Livers from thymectomized and control mice were dissected, enzymatically digested, and mechanically disrupted and stained with anti-CD45 (leukocytes), anti-CD3 (pan T cells), anti-CD4 (Helper T), anti-CD8 (Cytotoxic T), anti-CD44 (naïve/memory), and anti-CD62L (central/effector), anti-CCR2, anit-CCR5, and anti-CXCR3. **(A-C)** CD4+ T cell phenotype (naïve, central memory, and effector memory) and chemokine receptor expression in the spleen. **(D-F)** CD8+ T cell phenotype (naïve, central memory, and effector memory) and chemokine receptor expression in the spleen. N=8-10/group. Group differences assessed by unpaired t-test.

Supplemental Figure 8: T cell phenotype in the pgWAT. pgWATs from thymectomized and control mice were dissected, mechanically disrupted, enzymatically digested, and stained with anti-CD45 (leukocytes), anti-CD3 (pan T cells), anti-CD4 (Helper T), anti-CD8 (Cytotoxic T), anti-CD44 (naïve/memory), and anti-CD62L (central/effector). **(A)** proportion of leukocytes in the pgWAT. **(B)** Total number of Leukocytes per gram of tissue in the pgWAT. **(C)** Total number of CD3+ T cells per gram of tissue in the pgWAT. **(D)** Proportions of CD4+ Naïve, central memory (CM), and effector memory (EM) cells. **(E)** Proportions of CD8+ Naïve, central memory (CM), and effector memory (EM) cells. N=13-17/group. Group differences assessed by unpaired t-Test.

Supplemental Figure 9: T cell phenotype and chemokine receptor in the pgWAT. Livers from thymectomized and control mice were dissected, enzymatically digested, and mechanically disrupted and stained with anti-CD45 (leukocytes), anti-CD3 (pan T cells), anti-CD4 (Helper T), anti-CD8 (Cytotoxic T), anti-CD44 (naïve/memory), and anti-

CD62L (central/effector), anti-CCR2, anit-CCR5, and anti-CXCR3. **(A-C)** CD4+ T cell phenotype (naïve, central memory, and effector memory) and chemokine receptor expression in the spleen. **(D-F)** CD8+ T cell phenotype (naïve, central memory, and effector memory) and chemokine receptor expression in the spleen. N=8-10/group. Group differences assessed by unpaired t-test.

Supplemental Figure 10: Similarity between tissues and groups, differentially expressed gene count in the liver and pgWAT. (A) PCA analysis of tissue and group. **(B)** Number of significant DEG before adjusting for FDR. **(C)** Volcano plot showing Liver showing full DEG set prior to adjusting for FDR. **(D)** Volcano plot showing pgWAT showing full DEG set prior to adjusting for FDR. N=4/group.

Supplemental Figure 11: Gene Ontology terms in livers of thymectomized and control mice. (A) Volcano plot showing only significantly upregulated DEG after adjusting for FDR from the liver in thymectomized and control mice. **(B-C)** GO terms that are upregulated in livers of thymectomized or control mice. N=4/group. The P values were adjusted using the Benjamini & Hochberg method.

Supplemental Figure 12: Western Blot Images: (A) Full Image of FBP1 staining. **(B)** Ponceau S staining for total protein quantification.
