# Supplementary material for: Early-life thymectomy results in visceral adipose tissue inflammation and glucose intolerance
Source: Immun Ageing. 2025 Oct 1;22:36. doi: 10.1186/s12979-025-00531-x (PMC12487299; doi:10.1186/s12979-025-00531-x)

Supplemental Figure 1

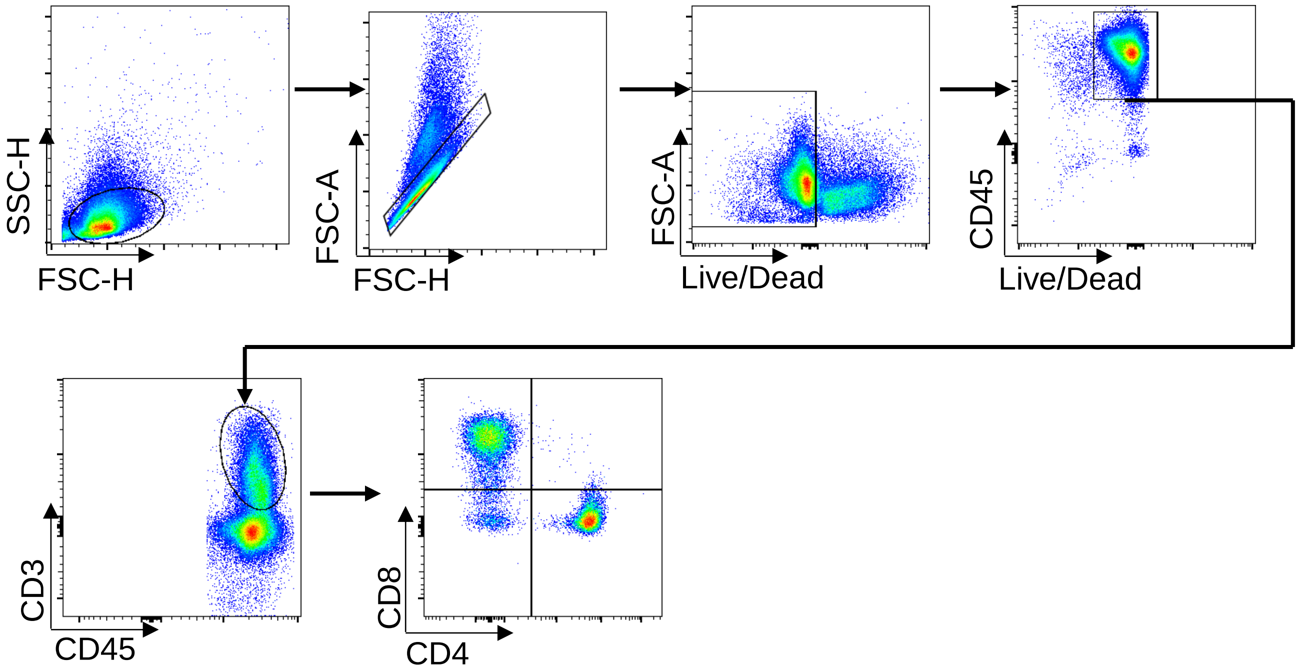

Phenotype and Chemokine Gating for CD4 and CD8

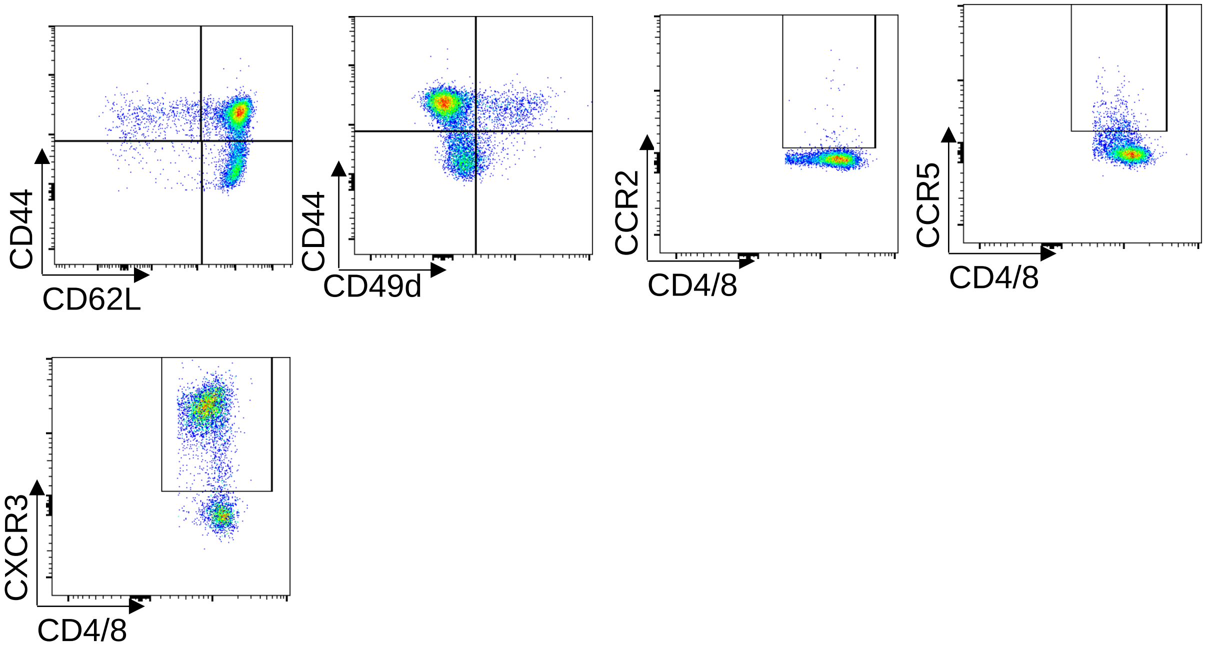

Supplemental Figure 2

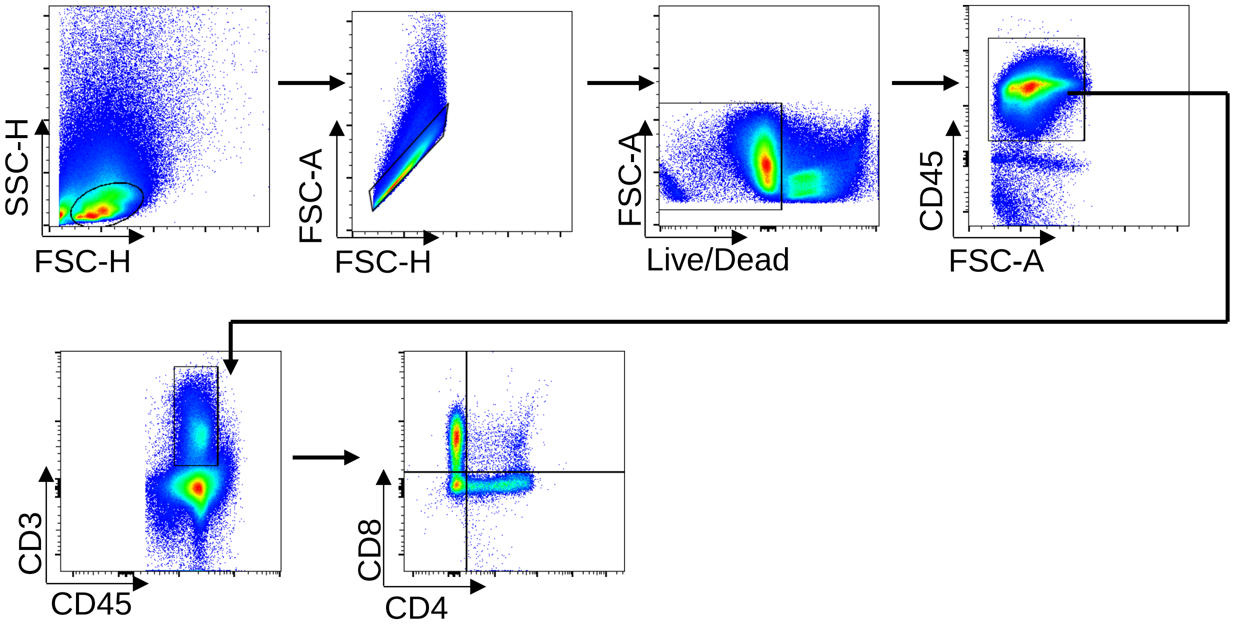

Phenotype and Chemokine Gating for CD4 and CD8

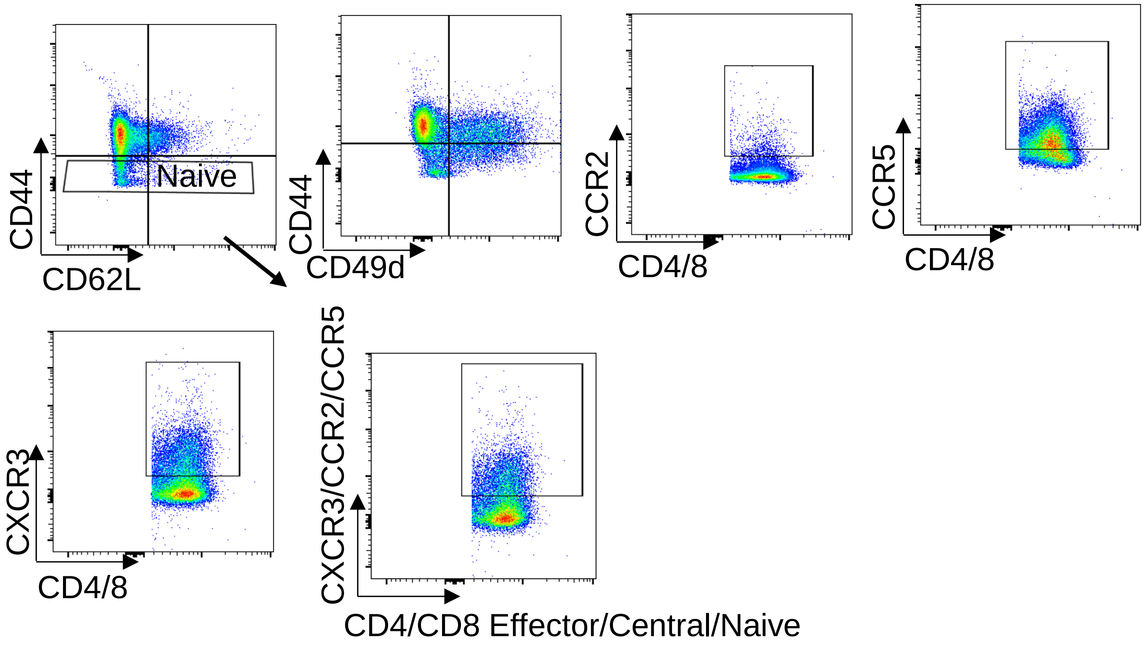

Supplemental Figure 3

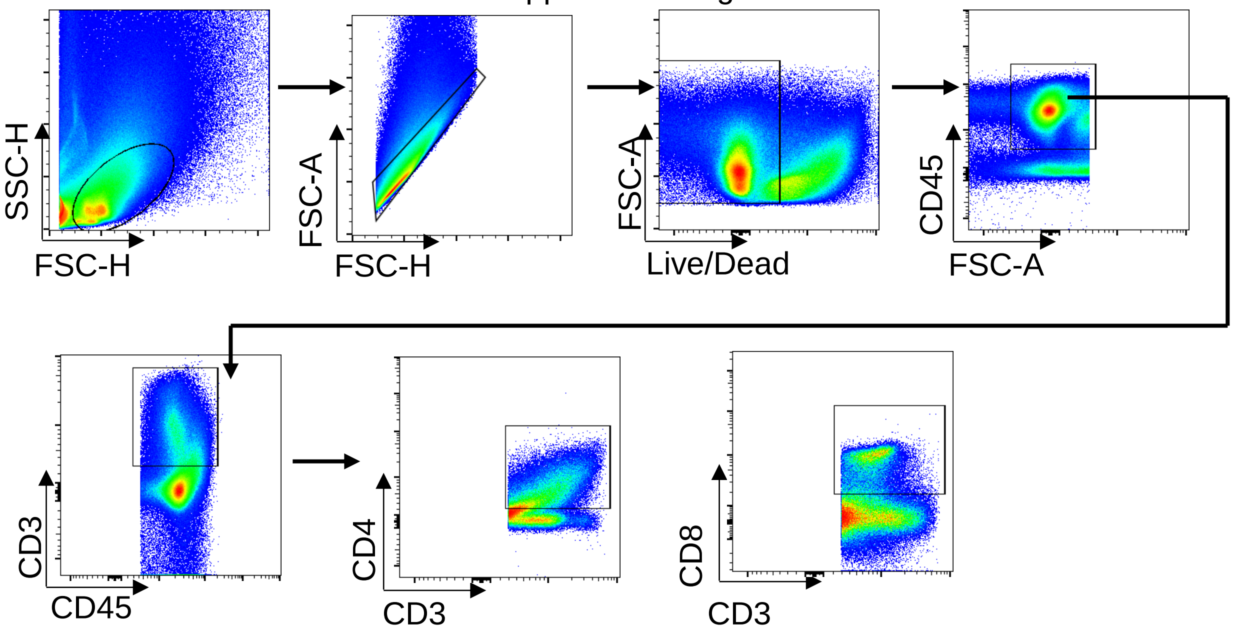

Phenotype and Chemokine Gating for CD4 and CD8

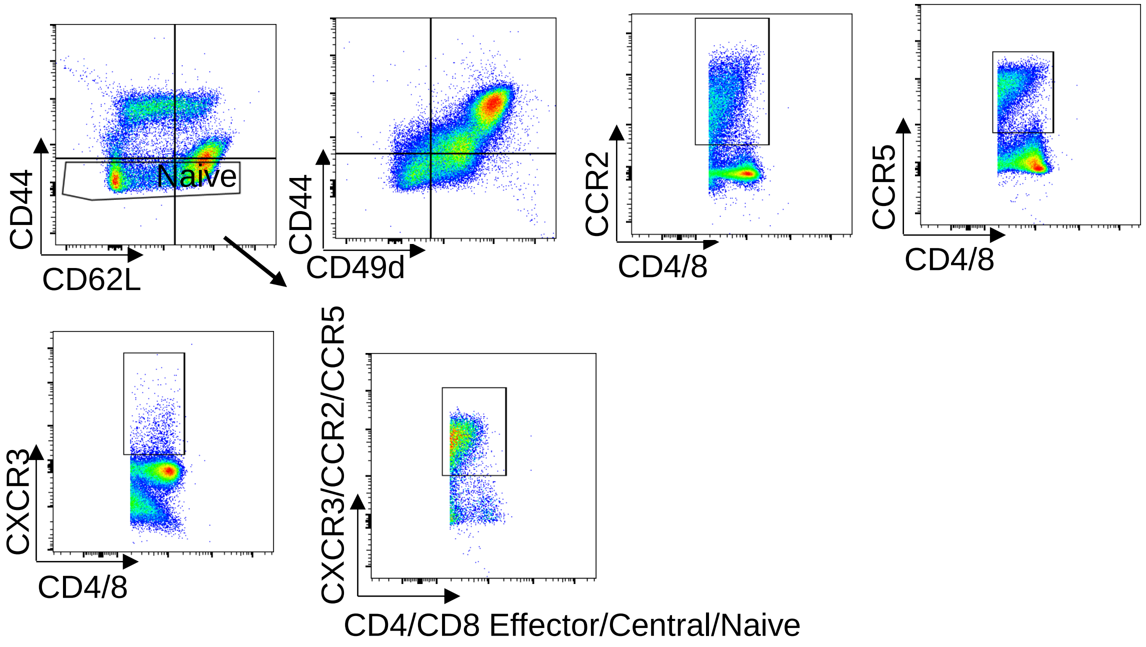

Supplemental Figure 4: Spleen

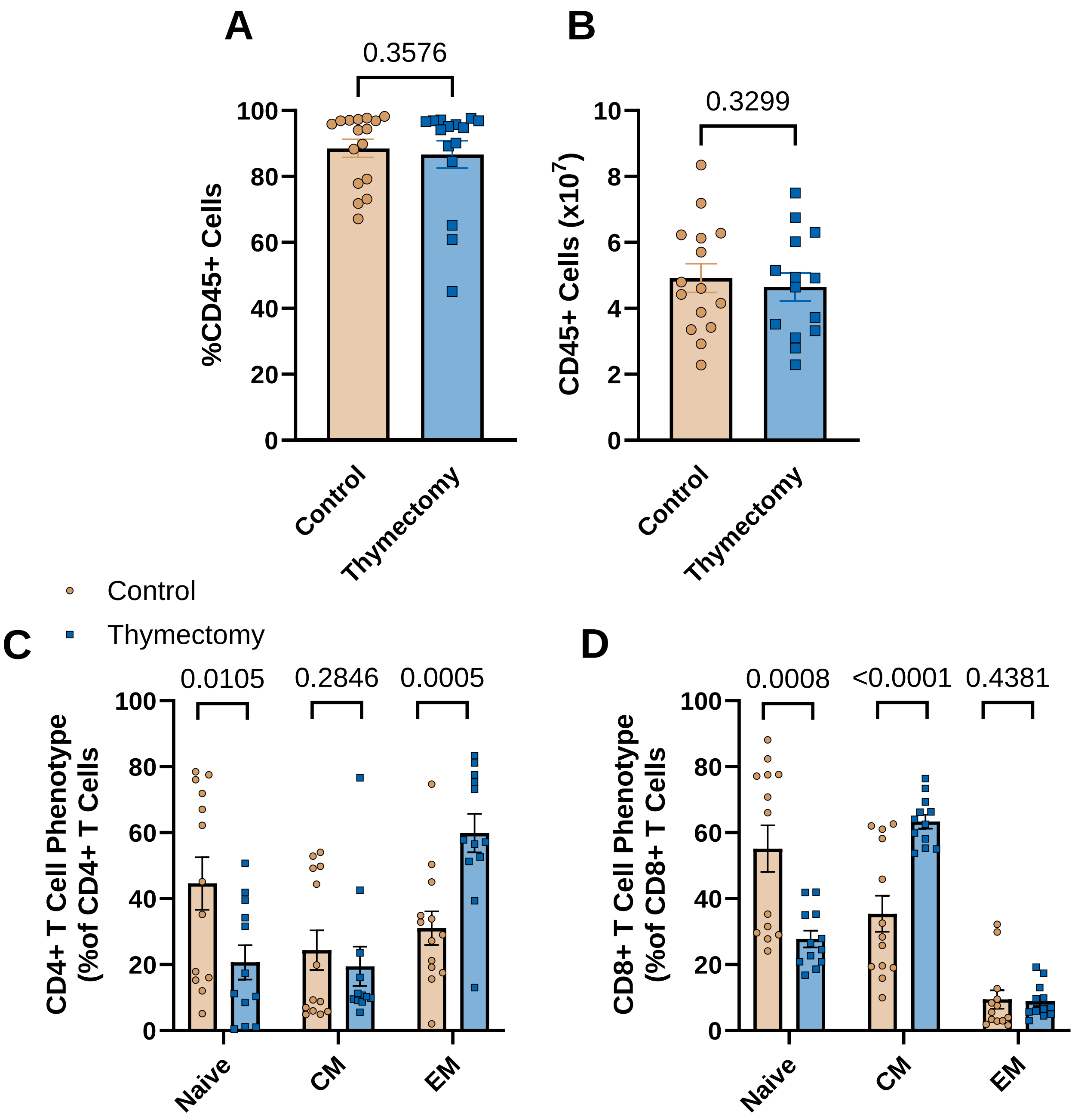

Supplemental Figure 5: Spleen

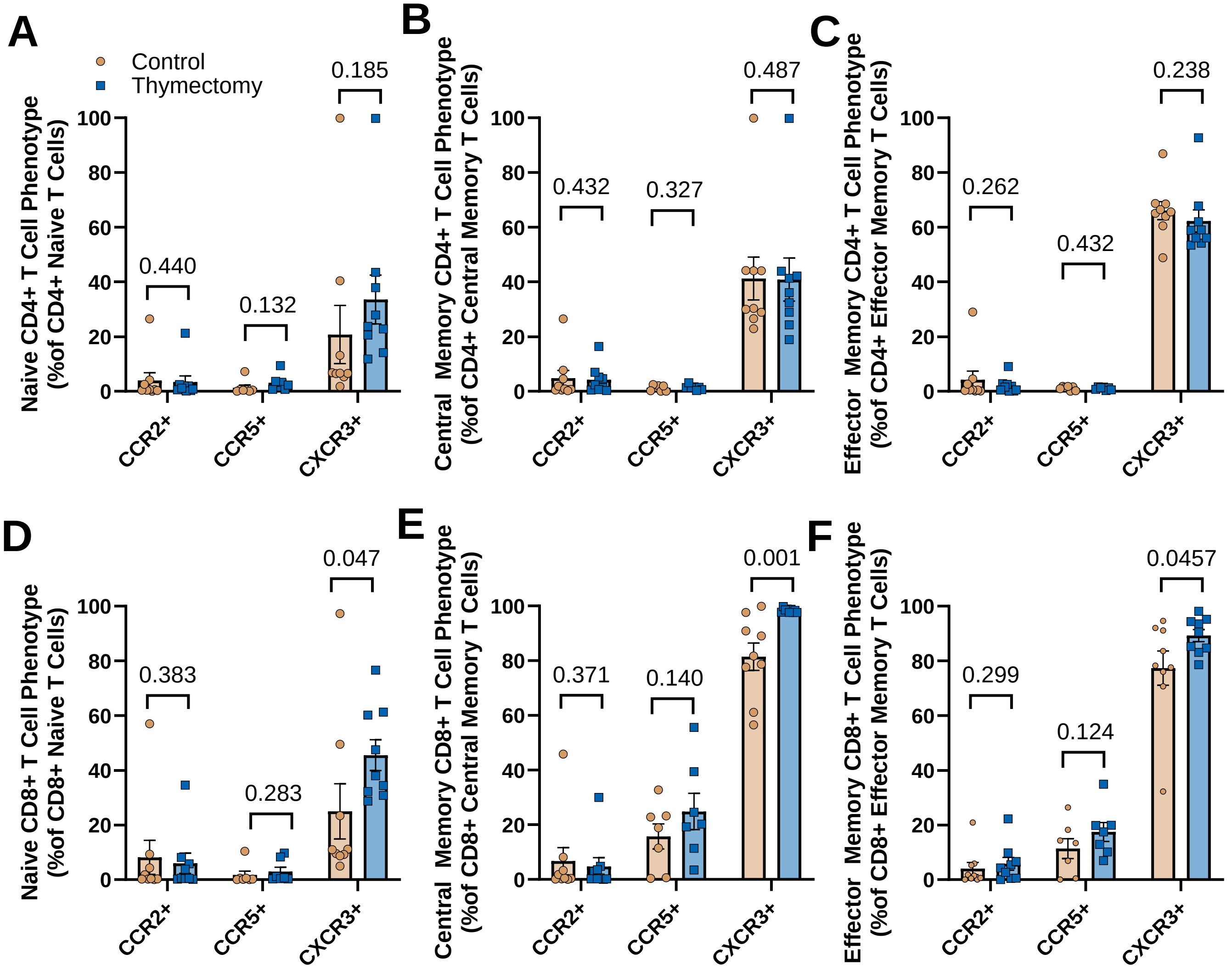

Supplemental Figure 6: Liver

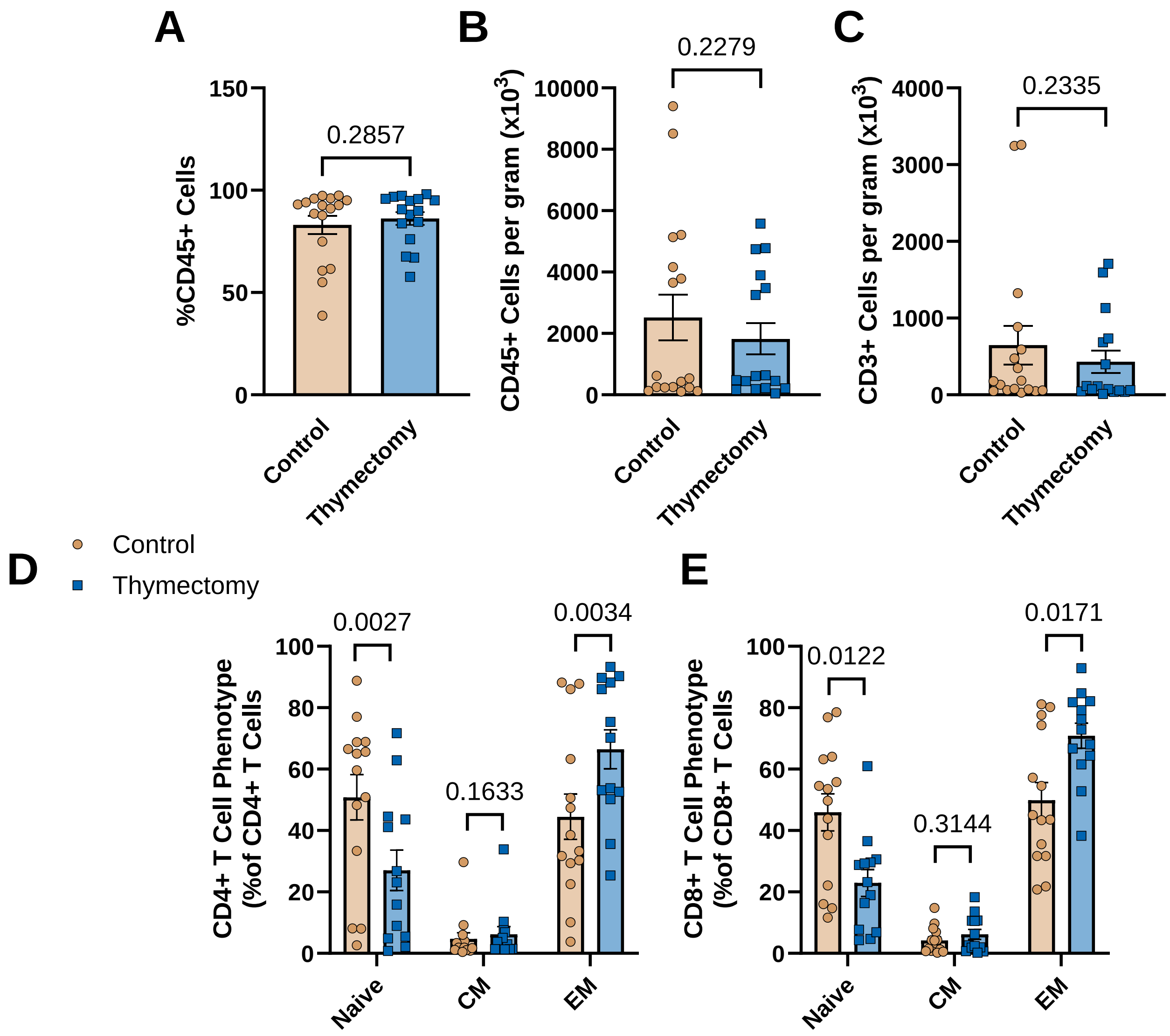

Supplemental Figure 7: Liver

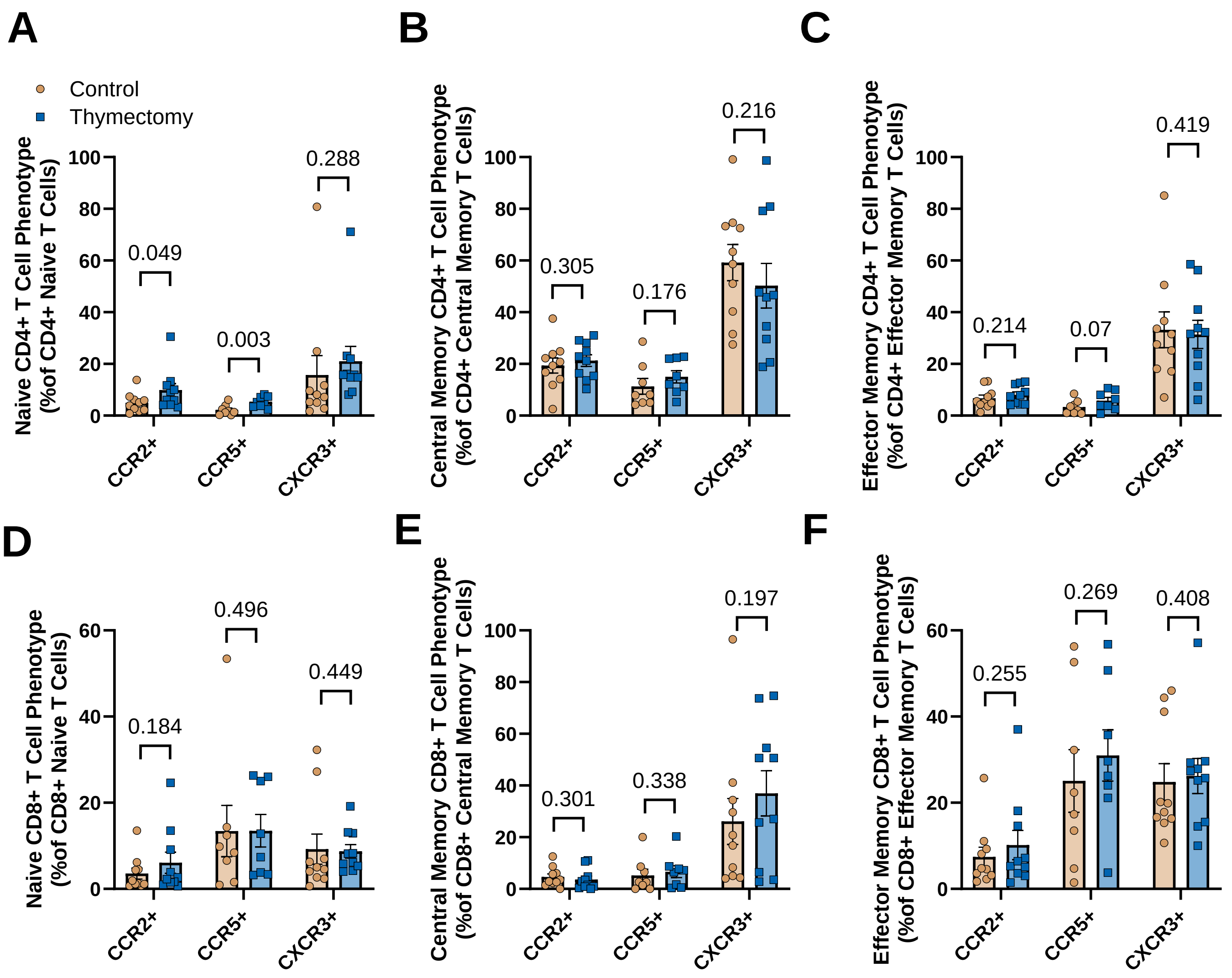

Supplemental Figure 8: pgWAT

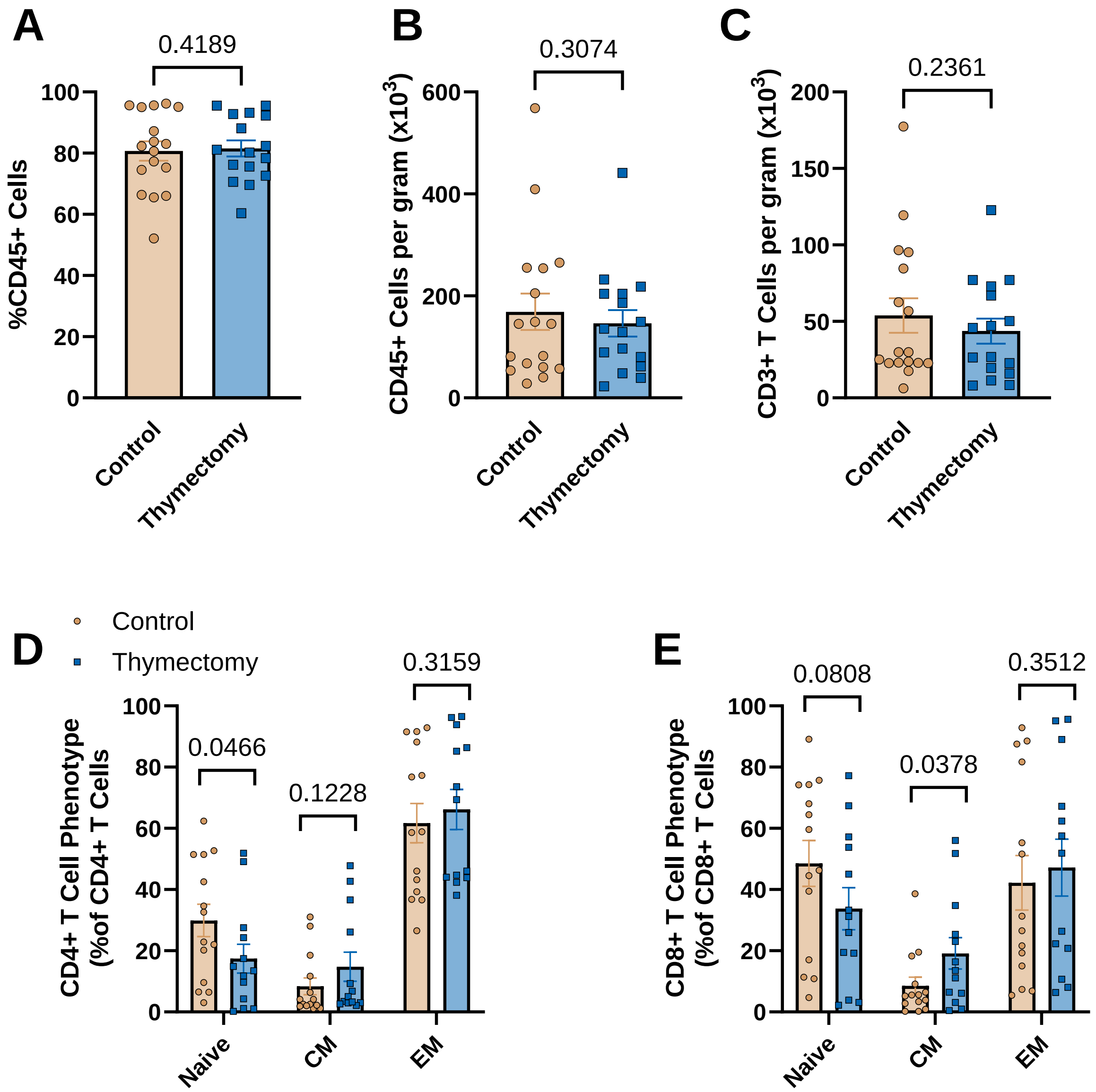

Supplemental Figure 9: pgWAT

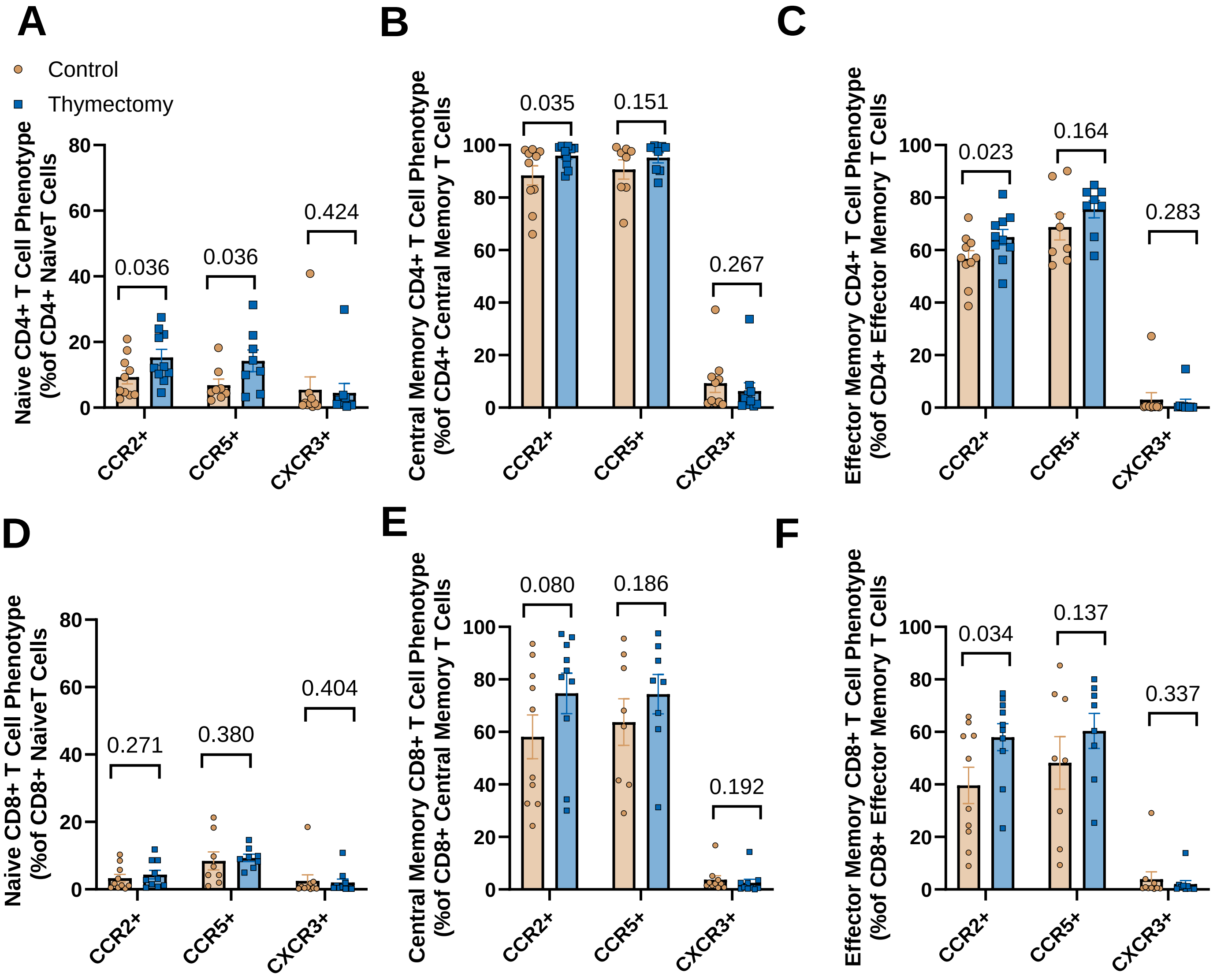

Supplemental Figure 10

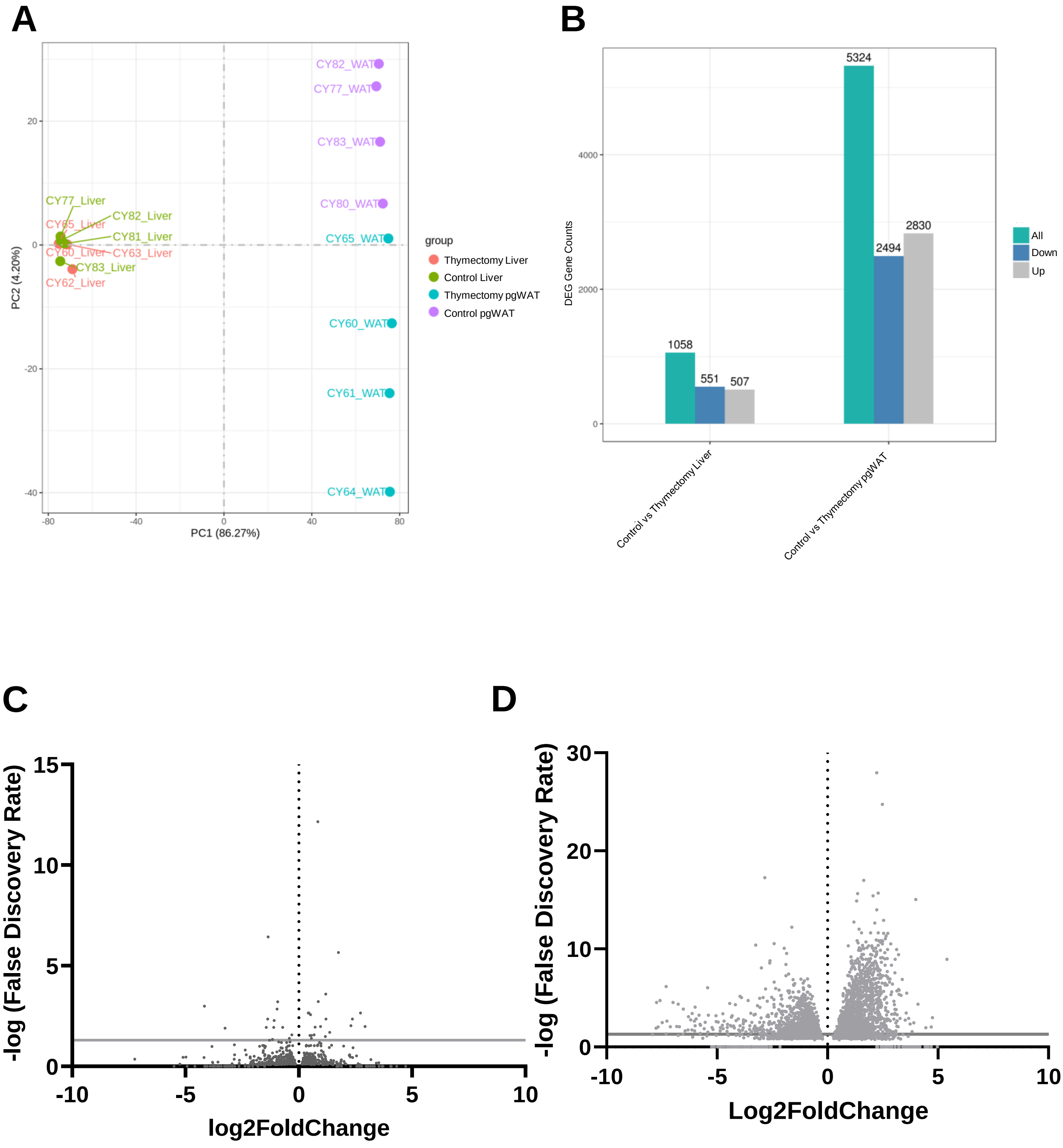

Supplemental Figure 11

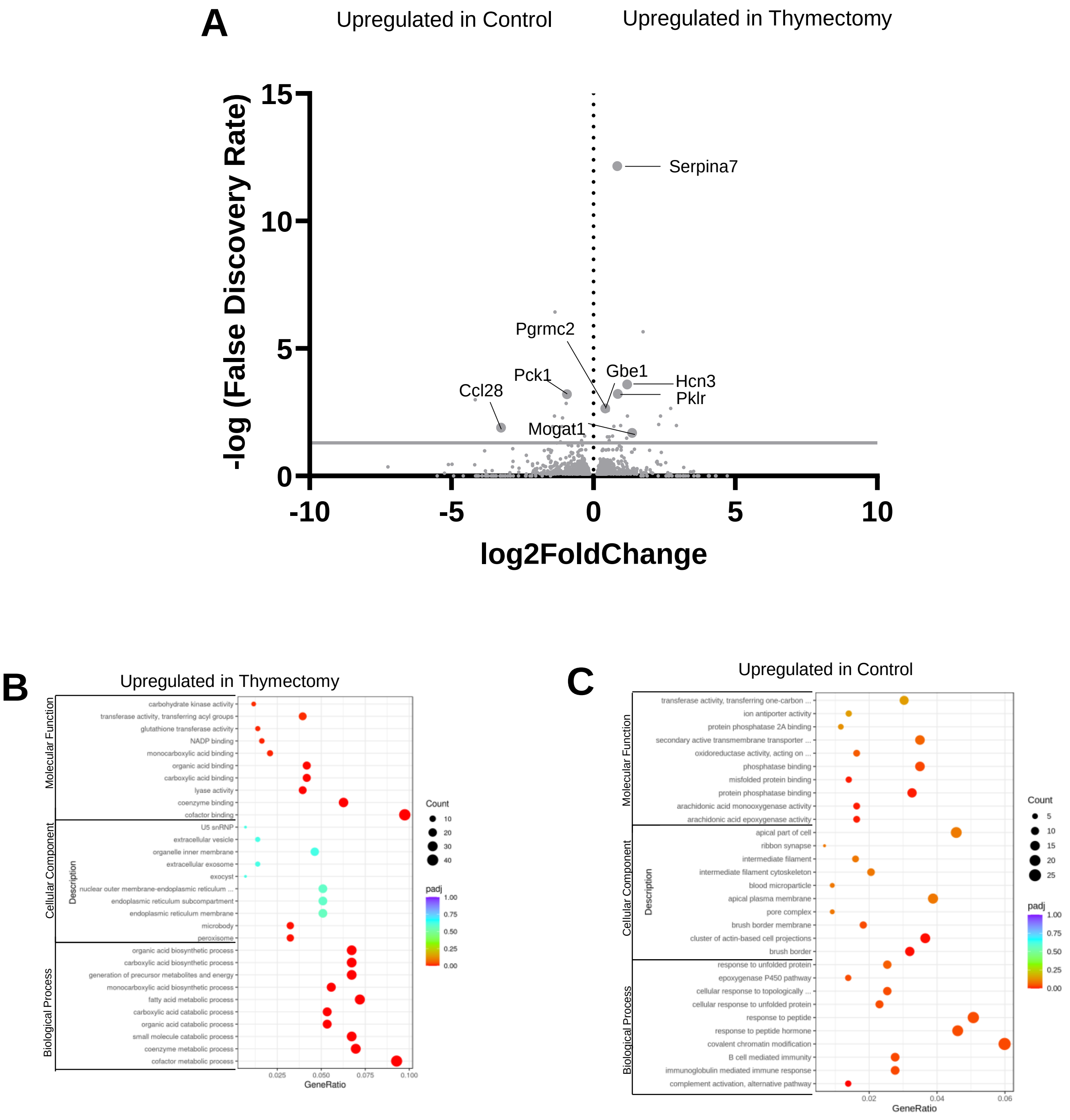

Supplemental Figure 12

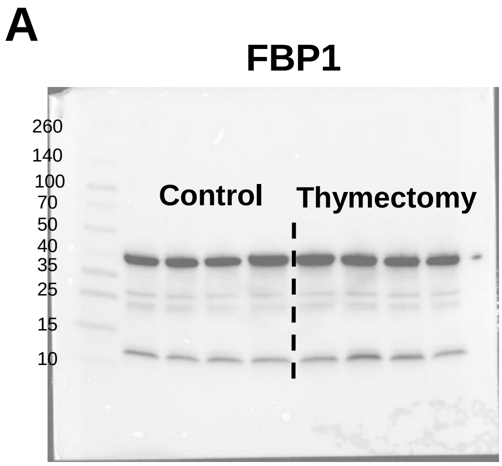

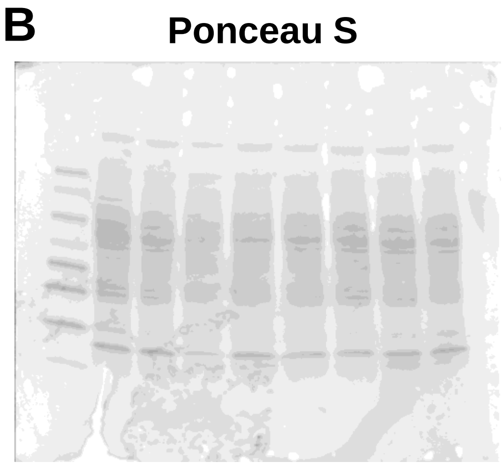

Supplement: Supplementary file 1 — Supplementary Material 1. [file 12979_2025_531_MOESM1_ESM.zip › Buckley et al supplemental material/Thymectomy Supplemental Figure Package Revision.pdf]
